# Water transit time and active recharge in the Sahel inferred by bomb-produced ^36^Cl

**DOI:** 10.1038/s41598-019-43514-x

**Published:** 2019-05-16

**Authors:** Camille Bouchez, Pierre Deschamps, Julio Goncalves, Bruno Hamelin, Abdallah Mahamat Nour, Christine Vallet-Coulomb, Florence Sylvestre

**Affiliations:** 1 Aix Marseille Univ, CNRS, IRD, INRA, Coll France, CEREGE, Aix en Provence, France; 2 Univ Rennes, CNRS, Géosciences Rennes, UMR 6118, 35000 Rennes, France; 3 University of N’Djamena, Département de Géologie, Ndjamena, Chad

**Keywords:** Element cycles, Climate-change mitigation, Hydrology

## Abstract

The lack of data and suitable methods to quantify regional hydrological processes often hinders sustainable water management and adaptation to climate change in semiarid regions, particularly in the Sahel, which is known for its climatic variability. Here we show that ^36^Cl from nuclear tests is a promising method to estimate water transit times and groundwater recharge rates on the catchment scale, and to distinguish water and chloride cycles. ^36^Cl was measured in 131 surface and groundwater samples in the Chari-Logone sub-catchment of the emblematic Lake Chad Basin, located in central Sahel. It was found that only 12 ± 8% of the catchment is connected to the main rivers. Groundwater supporting rivers in the upper humid part of the catchment has a mean transit time of 9.5 ± 1 years and a recharge rate of 240 ± 170 mm yr^−1^. In the lower Sahelian part of the catchment, stream-focused recharge yields recharge rates up to 78 ± 7 mm yr^−1^ in riparian groundwater against 16 ± 27 mm yr^−1^ elsewhere. Our estimates suggest that aquifers in the Sahel host a significant amount of renewable water, which could therefore be used as a strategic freshwater resource.

## Introduction

Freshwater resources in semi-arid regions are facing a number of stress factors, such as rapid population growth with the associated economic and agricultural developments, and climate change^1^. Concerns have been raised that growing pressure on freshwater resources might result in conflicts at sub-national to international levels. Sustainable water management relies on a sound understanding of fundamental hydrological catchment characteristics such as hydrologically active areas, catchment scale water transit times^2–4^ or groundwater recharge rates^5,6^. However, the assessment of these key parameters of the hydrological cycle remains difficult in semi-arid regions for two main reasons. First concepts are mainly adapted to temperate climates and thus can only be partly applied to these areas^7^, second hydrological and climatological data in many semi-arid and arid regions are scarce, particularly in sub-Saharan Africa^8^. The Sahel, a latitudinal belt stretching across the southern edge of Sahara, has been subject to humanitarian crises and social instability, exacerbated by the recurrence of persistent severe droughts since the 1980s^9^. Since then, standing debates have arisen on both the driving forces of climate variability in the Sahel^10^ and the complex non-linear hydrological responses to climate variability^11^. Despite international research programs^12^, Sahel’s catchment hydrology remains too poorly understood to support sustainable water governance. In particular, data on aquifer recharge rates and surface water – groundwater connectivity are lacking. Located in the centre of the Sahel, the Lake Chad Basin (LCB) is an endorheic catchment of 2.5 million km^2^. As Lake Chad integrates climatic and hydrologic changes over the region, it is a relevant scientific study site of Sahelian hydrology. The LCB is a hotspot of water-related issues in the Sahel, as the escalating conflicts over natural resources in Lake Chad point to the urgent need for a fair and sustainable management of natural resources.

Dissolved gas tracers (such as CFCs, ^3^H/^3^He, SF_6_, ^85^Kr) provide temporal constraints on the water transit time of surface and subsurface flows within a catchment^13^. However, contamination by atmospheric or soil gases^14^ often limits their applicability as age tracers to open-water systems. Bomb-produced tritium was widely applied as a young age tracer in the 1960s^6^, but the combined effect of removal by rain and radioactive decay (t_1/2_^3^H = 12.3 years) leads to an ambiguous age determination for recharge periods between 1975 and 2010^15,16^. ^36^Cl appears to be a relevant alternative tracer^17^: chloride is a ubiquitous ion, the production of ^36^Cl associated with nuclear tests reached three orders of magnitude above the natural ^36^Cl level^18^ and ^36^Cl is not affected by radioactive decay (t_1/2_^36^Cl = 301,000 years) for the time periods of interest. The nuclear ^36^Cl imprints in hydrological systems have therefore been used to estimate processes at a small scale in aquifers, soils or caves^19–22^ but, to our knowledge, have not been used to identify catchment characteristics on a regional scale.

Here, we developed an innovative approach based on ^36^Cl contents in all the components of the water cycle, to estimate regional-scale hydrological catchment characteristics in the LCB. First, the distribution of ^36^Cl in rivers, lakes and aquifers along a 500 km north-south transect was used to characterize both surface water and groundwater dynamics and infer their interactions. Second, from the combined study of the chloride and 36-chloride cycles, the hydrologically active proportion of the catchment was quantified and its mean transit time was evaluated. Third, the recharge rates of the aquifers were calculated.

### Hydrology of the Lake Chad Basin revealed by the spatial distribution of ^36^Cl

**Figure 1:**
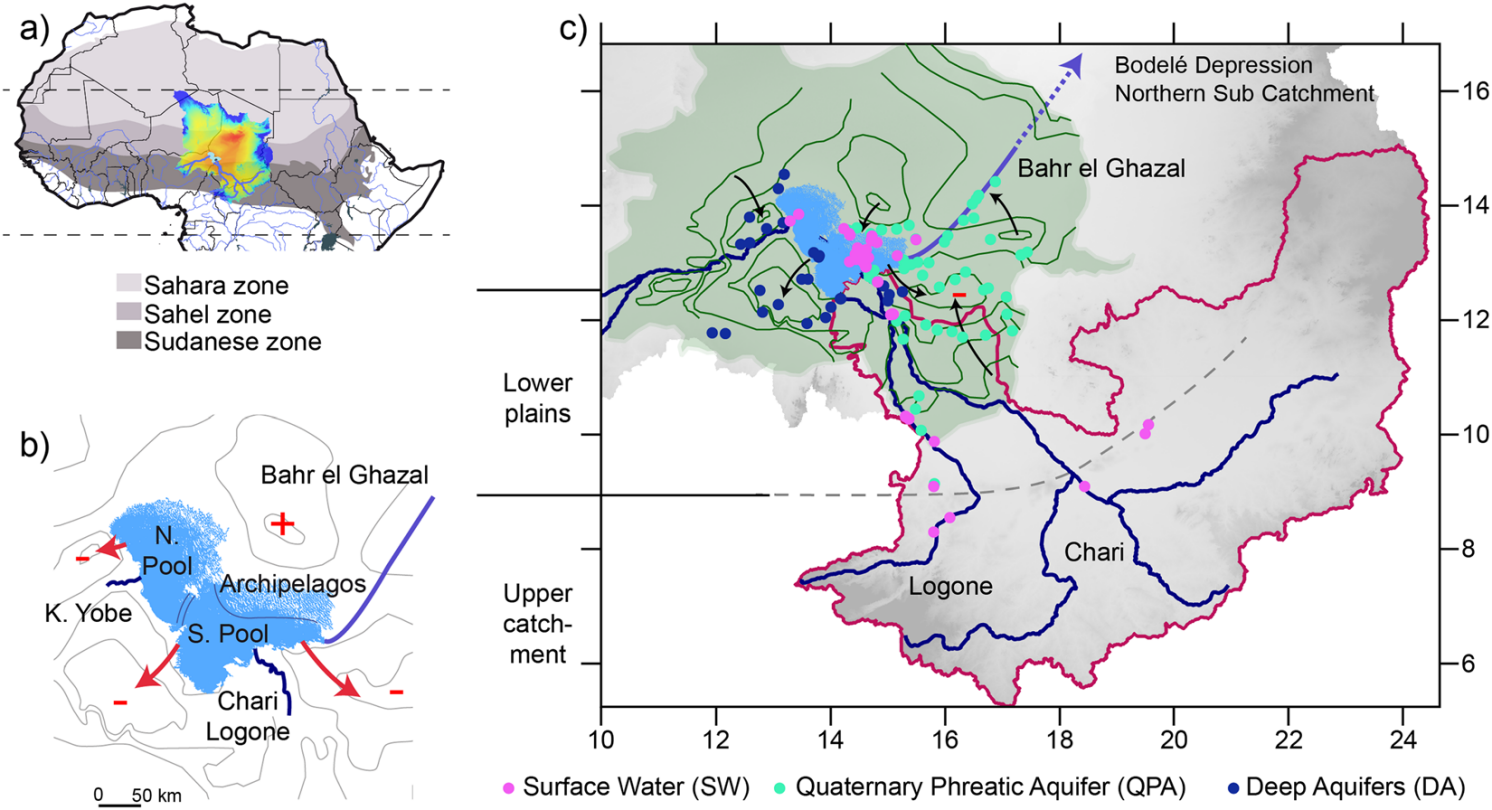
Lake Chad Basin location. (**a**) Extent of Lake Chad basin (2.5 million km^2^), coloured by elevation (dark blue >1000 m, dark red <200 m), with climatic zones and hydrological network. (**b**) Present-day Lake Chad (Southern Pool: S.Pool, Northern Pool: N.Pool and Archipelagos), main tributaries (Chari-Logone, Komadougou Yobe: K. Yobe) and present-day dry Bahr El Ghazal overflow channel. Arrows indicate flow directions. (**c**) Sampling locations (pink: Surface Water (SW), green: Quaternary Phreatic Aquifer (QPA), blue: Deep Aquifers (DA), Table 1 SI) with Chari-Logone catchment in red, Chari, Logone and Komadougou Yobe rivers in dark blue, Lake Chad in light blue, QPA extent and piezometric lines in green. Information on sampling points is available in Table S1.

The LCB is located in the centre of the Sahel and spans contrasting eco-climatic areas (Fig. 1), from south to north as a consequence of latitudinally decreasing rainfall. The Sudanese zone (~8°N–12°N) receives a mean annual rainfall between 1300 mm and 600 mm and is covered by dry forests and woody grasslands. The Sahelian zone (~10°N–16°N), with an annual rainfall between 600 and 200 mm, is dominated by grasslands and the Saharan zone (~16°N–30°N) is a desert with rainfall below 200 mm/yr^23^. Its hydrology is dominated by the Chari-Logone river that drains water from the southern humid Sudanese zones into the endorheic Lake Chad^24^ (Fig. 1). Lake Chad is very sensitive to climatic changes as its surface decreased from 25,000 km^2^ to 4,000 km^2^ during the severe drought that affected the Sahel in the 1980s^25^. Groundwater occurs in saprolite aquifers in the Sudanese upstream part of the catchment (referred to as UCSA) and in large sedimentary aquifers in the Sahelian downstream part. From the deepest to the shallowest, the main downstream aquifers are the confined Continental Terminal and Pliocene aquifers (referred to as Deep Aquifers, DA) and the shallow Quaternary phreatic aquifer (referred to as QPA) (Fig. S-A).

One hundred and thirty-five samples of rainfall, rivers, Lake Chad waters and groundwater of the QPA and DA were collected over the Chari-Logone catchment, a sub-catchment of the Lake Chad Basin, and analysed for ^36^Cl, δ^18^O, δ^2^H and major elements (*S*.*M*. *2 for details on the hydrogeological settings*, *sampling points*, *chemical and isotopic analyses and raw data*). Groundwater samples with high concentrations in both Cl^−^ and NO_3_^−^ could be indicative of human pollution by agricultural or industrial inputs, and were therefore excluded from the following results (8/71 samples, *S*.*M*. *2*.*2 -* Table S1 - Fig. S-B).

**Figure 2:**
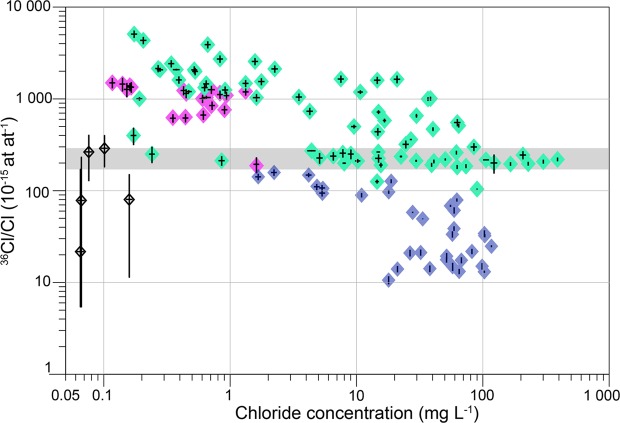
Distributions of ^36^Cl/Cl ratios measured in waters of the Lake Chad Basin. ^36^Cl/Cl (x10^−15^ at at^−1^) ratios as a function of chloride concentration (mg L^−1^) with analytical uncertainties (1-sigma), measured in all water components of the LCB: rainfall (black), surface waters (pink), QPA (green) and DA (blue). The ^36^Cl/Cl natural atmospheric input for the region (216 ± 23 × 10^−15^ at at^−1^, see calculations SI section 4.1) is represented by a grey band. Raw data are available in Table S1.

A large range of ^36^Cl/Cl ratios (10–5000 × 10^−15^ at at^−1^) and chloride concentrations (0.1–300 mg L^−1^) were measured (Fig. 2). In the DA, ^36^Cl/Cl ratios did not exceed 150 × 10^−15^ at at^−1^ while shallow QPA groundwater and surface waters had similar ranges of ^36^Cl/Cl ratios, between 200 and 5000 × 10^−15^ at at^−1^. DA groundwater also had significantly more depleted δ^18^O and δ^2^H compositions than QPA groundwater and surface waters (Fig. S-C). The low ^36^Cl/Cl ratios (down to 10 × 10^−15^ at at^−1^) and depleted δ^18^O values in the DA water samples mark old groundwater recharged during previous humid periods. Based on the radioactive decay of ^36^Cl, groundwater ages up to 1 Ma were suggested in the deep aquifers^26^.

QPA groundwaters have ^36^Cl/Cl ratios between 100 and 5000 × 10^−15^ at at^−1^. A constant ^36^Cl/Cl ratio of 216 ± 23 × 10^−15^, independently of the chloride concentration, was measured in a large number of samples located all across the QPA (n = 25, Fig. 2). It corresponds to the natural ^36^Cl/Cl atmospheric input in this region, from which a natural ^36^Cl fallout of 9 ± 5 at m^−2^ s^−1^ can be calculated (*S*.*M*. 3.2). This value is consistent with ^36^Cl fallout latitude-dependent modelling yielding 10 at m^−2^ s^−1^ in the 10 to 20°N latitudinal band^17^. Only two groundwater samples in the QPA have a ^36^Cl/Cl ratio lower than the natural atmospheric ratio, showing that halite dissolution is not a major source of chloride in the LCB. QPA groundwaters with ^36^Cl/Cl ratios above the natural atmospheric ratio trace an anthropogenic source of ^36^Cl linked to nuclear tests performed in the 1960s. QPA waters that contain bomb-produced ^36^Cl mark a recharge during the last 60 years, commonly referred to as modern groundwaters^6^. Conversely, and because present-day surface waters still show ratios above the natural atmospheric ratio, groundwater samples that exhibit ^36^Cl/Cl ratios within the natural background level mark a recharge prior to the nuclear tests and thus older than 60 years.

The age but also the origin of the recharge of the QPA can be inferred from combining ^36^Cl data with δ^18^O data, since as a result of the progressive evaporation of water flowing towards the terminal Lake Chad, the surface waters show a wide range of δ^18^O compositions (δ^18^O rainfall = −4‰, δ^18^O river = −3‰, δ^18^O lake = 2 − 10‰). Modern groundwaters (^36^Cl-enriched) are found in the vicinity of the Chari-Logone river and Lake Chad (δ^18^O-enriched), showing that focused recharge tied to the surface hydrological network dominates in the QPA, as expected in semi-arid environments^7^ (Fig. S-D). Pre-modern groundwaters (^36^Cl-background) can be classified into δ^18^O -enriched samples (δ^18^O > −2‰) and δ^18^O -depleted samples (δ^18^O < −2‰). δ^18^O -enriched pre-modern groundwaters are located around the Bahr El Ghazal (Fig. 1), an overflow channel of Lake Chad, active only during high levels of the lake (h > 283 m, latest overflow in the 1950s). High-level Lake Chad stages therefore increase recharge of the QPA. δ^18^O –depleted pre-modern groundwaters, characterized by higher total dissolved solutes (EC > 1500 μS cm^−1^), are located in piezometric depressions, which are common but poorly understood hydrogeological features throughout the Sahel^27,28^. ^36^Cl/Cl ratios similar to present-day background ratios and ^14^C contents above 50 pmc (*S*.*M*. *5*.*3*) also point to Holocene waters^29^ Therefore, groundwaters of the piezometric depressions are likely related to the replenishment of Sahelian aquifers during the African Humid Period^30^, from 12,000 to 5,000 years ago^31^.

Present-day surface waters depicted enriched ^36^Cl/Cl ratios, while rainfall ratios fell to around the natural ^36^Cl/Cl ratio, even if the restricted rainfall sampling might not be fully representative of the ^36^Cl annual variability^32^ (Fig. 2). The presence of nuclear ^36^Cl in surface waters and not in rainfall highlighted an input of long-term chloride, carrying anthropogenic ^36^Cl, during water transfer through the subsurface. The upper Chari-Logone, the lower Chari-Logone rivers and the Southern Pool of Lake Chad (Fig. 1) had similar ^36^Cl signatures (Table S1), suggesting that anthropogenic ^36^Cl entered the headwaters in the Sudanese part of the LCB and was preserved downstream. The Komadougou Yobe river, which drains the Sahelian region between Niger and Nigeria (Fig. 1), had less ^36^Cl-enriched waters than the Chari-Logone, revealing different water dynamics between these two sub-catchments (Table S1).

The ^36^Cl data gathered on LCB waters show that shallow aquifers are relevant objects to determine the ^36^Cl/Cl regional natural background, as they integrate spatial and temporal variations of ^36^Cl in rainfall^32^. Data show that the upstream Sudanian reach of the Chari-Logone river is gaining and received input from ^36^Cl-enriched subsurface waters, while the downstream Sahelian reach of the Chari-Logone is losing and recharges the QPA.

### Quantification of the present-day hydrologically active surface of the Lake Chad Basin

The chloride mass balance was calculated under the assumption that anthropogenic Cl sources were negligible compared to natural Cl deposition. The chloride export through the Chari-Logone (6.9 ± 3.4 × 10^6^ kg yr^−1^, *S*.*M*. *2*.*3*) corresponds to 12 ± 8% of the chloride that was deposited on the total surface of the Chari-Logone catchment (1 ± 0.2 kg yr^−1^ ha^−1^ × 600 000 km^2^ = 6 ± 1 × 10^7^ kg yr^−1^, *S*.*M*. *3*.*1 and 4*.*3*). The majority of Cl deposition was thus not exported through the Chari-Logone river, indicating that a large portion of deposited Cl accumulates in the basin because most of the rainfall is intercepted before reaching the main river. Several climatic and geomorphological features of the catchment could explain this large discrepancy between precipitation and river flows. First, this region is characterized by very high evaporation rates (1300–2000 mm yr^−1^). Second, long distances to the closest drain, and low slopes of the catchment landscape, could exacerbate water evapotranspiration. Third, because the DA only outcrops in the south of the catchment, some of the rainfall is likely to recharge this aquifer and therefore not contribute to the surface water cycle.

The chloride deficit between deposition and river export was as high as 88 ± 8%, suggesting that only 12 ± 8% of the catchment is hydrologically connected with the Chari-Logone network. Over this connected part of the catchment, a runoff coefficient of 29 ± 20% was calculated based on the downstream runoff and a rainfall rate of 1350 ± 200 mm yr^−1^, which corresponds to the average rainfall over the 12 ± 8% most humid part of the catchment. Therefore, the previous estimate of a 7% runoff coefficient^33^ over the entire basin is misleading as it neglects climatic heterogeneity. Here we distinguished between disconnected areas, with a 0% runoff coefficient and where salt accumulates, from connected areas, with a positive runoff coefficient and where chloride is transported to the river. We determined a theoretical precipitation over potential-evapotranspiration ratio threshold of 0.95 (*S*.*M*. *4*.*3*), below which an area is considered to be disconnected from the main river and chloride accumulates either in soils or aquifers or is exported by eolian transport. This value is higher than the threshold values typically encountered in arid regions^34^ but since it is close to unity, it indicates that the hydrology of this catchment is very sensitive to changes in precipitation. The large proportion of disconnected areas together with the strong control of climate on surface hydrology might yield rapid connections or disconnections of sub-catchments. This could be one explanation for the non-linear dynamics of Sahelian catchments observed previously^11^. It also suggests that in arid regions, the chemical memory effects of catchments are likely to be relatively important^35^.

### Determination of transit time in the upper Sudanian sub-catchment

**Figure 3:**
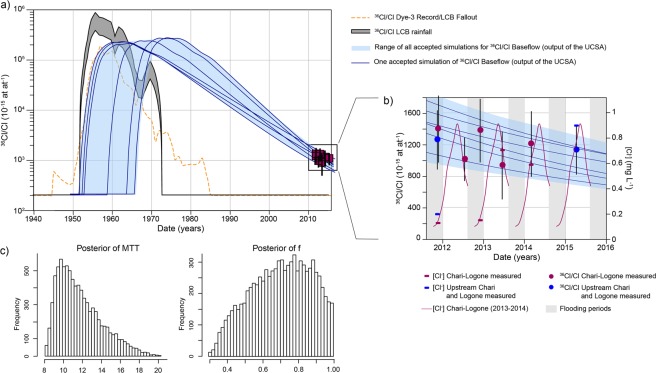
Mean transit time in the saprolite aquifer (UCSA), located in the Sudanese zone and supporting baseflow of the Chari Logone in the Sahelian zone. (**a**) Measured ^36^Cl/Cl in the Chari-Logone (red squares), ^36^Cl/Cl range in rainfall (gray band) – calculated from simulated ^36^Cl deposition^39^ and calculated Cl depositions on the LCB, ^36^Cl/Cl measured at Dye-3^51^ (orange dotted line), simulated ^36^Cl/Cl of baseflow (=output of the bedrock aquifer): envelope of all simulations in pale blue and individual realizations of the EPM (with different MTT and f) in dark blue lines. (**b**) Measured (purple and blue dots) and simulated (envelope in pale blue and individual simulations in dark blue lines) ^36^Cl/Cl in the Chari-Logone, and measured [Cl-] (purple lines) in the Chari-Logone between 2011–2015. Data are available in Table S2 and calculations are fully described in the SM. Error bars on measured ^36^Cl/Cl account for both analytical errors and uncertainties of the baseflow proportion estimate. (**c**) Posterior distributions of parameters MTT and f calculated from accepted parameters of the MCMC (15%).

The ^36^Cl/Cl ratio measured in the Chari-Logone river decreased over the sampling period, which can be attributed to a progressive dilution of the bomb-produced ^36^Cl^18^ (Fig. 3). The measured ^36^Cl/Cl ratios were used to determine the water transit time in the connected part of the Chari-Logone catchment, where water transit time can be estimated from chloride transit time, as chloride does not accumulate in this part of the catchment. To do so, the ^36^Cl/Cl ratio in the Chari-Logone headwaters was simulated between 1956 and 2015, by a mixing model between surface runoff and subsurface runoff through the UCSA (*S*.*M*. *4*.*1 and 4*.*2*). The mixing proportion was determined via three methods, Na^+^ mass balance^36^, δ^18^O amplitude ratio^2^ and hydrograph separation^37^. The ^36^Cl/Cl time series in surface runoff was taken equal to the ^36^Cl/Cl time series of rainfall. The ^36^Cl/Cl time series in the baseflow was simulated by convoluting the ^36^Cl/Cl time series of rainfall with an Exponential-Piston flow model (EPM) with two parameters, the mean transit time (MTT) and the ratio of exponential to piston flow (f) (*SM section 4*.*2*). The choice of the EPM to represent the transit time distribution within the aquifer was motivated by the geometry of an unconfined aquifer sampled at its outlet (in the river)^38^. The ^36^Cl/Cl ratio of rainfall between 1950 and 2015 was obtained from worldwide simulations of bomb-produced ^36^Cl deposition^39^ divided by atmospheric chloride deposit over the catchment (*SM section 3*.*1 and 3*.*4*). Combined uncertainties of ^36^Cl and Cl depositions yield a range of ^36^Cl/Cl time series of rainfall (Fig. 3). Oriented Markov Chain Monte Carlo procedures using a Metropolis-Hasting algorithm (n = 10 000 simulations) were implemented with ten input ^36^Cl/Cl rainfall series, uniformly distributed within the calculated range of ^36^Cl/Cl time series in rainfall. Analytical uncertainties on observed ^36^Cl/Cl in the Chari-Logone river were taken into account in the calculation of the lognormal likelihood function of the Metropolis-Hasting algorithm^40^. Posterior distributions of the two parameters (MTT and f) were calculated based on the accepted set of parameters of all Metropolis-Hasting runs. Therefore, the distributions of parameters reflect the deviation which would still match ^36^Cl/Cl input of rainfall within its uncertainty, as well as the measured ^36^Cl/Cl ratios within their standard analytical error.

The annual subsurface runoff accounted for 60% (±10%) of the Chari-Logone discharge and for 70% (±10%) of Cl inputs. MTT of 9.5 ± 2 years and a proportion of exponential flows of 0.8 ± 0.2 were estimated (Fig. 3). Consistently with previous studies of catchments with a MTT below 40 years^15^, the model was found less sensitive to the f parameter than to the MTT, which is supported by the higher standard deviation of f compared to MTT. Simulations matched the observed ^36^Cl/Cl ratios and their decreasing trend (Fig. 3). However, the same calibration procedure using other models yielded MTT ranging between 2 and 12 years, with a maximum of likelihood around 3 years (*S*.*M*. *4*.*3*). The estimated MTT might therefore be overestimated by the EPM model. As the productive hydrological area is restricted to a small sub-catchment, uncertainties associated with aggregation in the determination of MTT are likely to be limited under the present climatic conditions. However, if the catchment wets up, the contribution of groundwater from other sub-catchments is likely to cause aggregation effects yielding large errors in the estimation of the MTT^2,41^. High flows were slightly more enriched in ^36^Cl than low flows, although this difference is within the range of uncertainties associated with the lumped-parameter model. At low flows, ^36^Cl could be immobilized and subsequently remobilized at high flows. A seasonal retention of Cl could therefore occur in the productive basin, but may be buffered at an annual time step, which is consistent with short flows in the unsaturated zone suggested by the small piston flow proportion parameter (0.2)^15,38^.

### Quantification of groundwater recharge in the upstream Sudanian part and the downstream Sahelian part

Based on the water, Cl and ^36^Cl budgets, we inferred that the hydrologically active surface was restricted to 12 ± 8% (70 000 km^2^) of the total surface of the catchment with a total runoff coefficient of 29 ± 20%, 60 ± 10% of which transited through the UCSA with a mean transit time of 9.5 ± 1 years. Therefore, the UCSA is characterised by a recharge rate of 240 ± 170 mm yr^−1^ (60% of 29% of 1350 mm) and an equivalent depth of groundwater stored of 2.3 ± 1.6 m (0.24 * 9.5), consistently with global estimates of modern groundwater volumes^6^.

**Figure 4:**
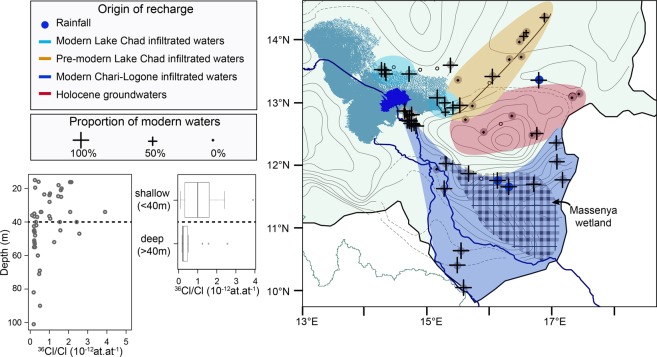
Map of the modern recharge in the Quaternary Aquifer of the Lake Chad Basin. The map synthetises the location and the source of the recharge of the quaternary aquifer in the downstream Chari-Logone basin. The proportion of modern water is represented by black crosses. The source of the infiltrated waters (inferred from ^36^Cl/Cl ratios and δ^18^O) is displayed with coloured zones. Black circles show polluted samples. See SM for full description of the calculations.

In the Sahelian part of the catchment, the ^36^Cl and Cl distributions in groundwaters of the QPA were interpreted by a binary mixing between old groundwater and modern groundwater, affected by evaporation (Fig. S-F). On average, waters recharged since 1952 account for 94 ± 8% of groundwater in the vicinity of the hydrological network and only 19 ± 32% further away, which confirms that recharge derives mainly from nearby streams in the Sahelian part of the catchment (Fig. 4). Modern groundwaters were only found at shallow depth (<40 m), which corresponds to the upper half of the aquifer (Fig. 4). A present-day annual renewal rate of 0.74 ± 0.06% yr^−1^ (0.94 * 0.5/(2015–1952)) and a recharge rate of 78 ± 7 mm yr^−1^ can be derived for groundwater close to the hydrological network (using mean saturated thickness and porosity of respectively 35 m and 0.3). However far from the hydrological network, recharge is much lower and we estimate a present day annual renewal rate of 0.1 ± 0.3% yr^−1^ and a recharge rate of 16 ± 27 mm yr^−1^. Recharge estimates are higher than present-day withdrawal rates out of the QPA^42^.

### Sahelian water resources

Mean transit time in a complex hydrogeological system provides a first indication about its potential to buffer hydrologic variability^43^. The Sahel shows quite homogeneous climatic and geologic settings and its hydrology is strongly controlled by the humid zones of equatorial Africa (here named the Sudanese zone). The decadal mean transit time and the low storage capability of the basement aquifer providing baseflow demonstrate the vulnerability of Sahelian surface waters to potential climate or land-use changes in the tropical humid zone. Sahelian aquifers showed a present day recharge restricted to the south of the catchment and the near-surface hydrological network. However, the calculated renewal rate and the net infiltration rates suggested that Sahelian aquifers host a significant amount of water inherited mainly from past humid periods but still recharged presently. Most of the Sahelian population resides in the south where one of the highest population growth rates in the world is recorded. In the South of the Chari-Logone, 100% of irrigation water is supplied by surface water, and 65% in the North^42^, while surface waters are vulnerable. On the other hand, Sahelian aquifers are not over-exploited, as recharge is higher than extraction rates, contrary to most aquifers in the world^44^. Therefore, if adequate infrastructures are developed and strong regulation policies are adapted to prevent depletion^44^, Sahelian aquifers could act as a strategic freshwater resource, mitigating the high pressure on water resources in the area.

The present study demonstrates the suitability of ^36^Cl to quantify the regional hydrological cycle and, combined with Cl to distinguish between the chloride and water cycles. Our data suggest that a large proportion of the catchment is presently disconnected from the main drainage network. However, the present approach integrates processes over long time periods, from years to decades, and neglects short temporal variability, while extreme events were shown to be major drivers of groundwater recharge^45,46^. Extreme events might also have the potential to reconnect formerly disconnected parts of the catchment, with unknown consequences for water quality and water residence time distributions. As climate models predict higher frequency and intensity of extreme rain in the Sahel^47^, future studies should aim at gathering high-frequency data to evaluate the transient dynamic of catchment hydrology in the Sahel.

## Methods

Measurements of ^36^Cl were carried out by Accelerator Mass Spectrometry^48^ at the French AMS National facility, ASTER at CEREGE^49^. Because of low chloride contents in surface and some groundwater samples, the addition of a ^35^Cl enriched spike was required to reach a total chloride amount of 2 mg and to precisely determine the Cl concentration using the ID-AMS technique^50^. Water samples were processed in batches of 10–15 samples with a spiked blank and an unspiked blank at the end of each batch to estimate chemical contaminations on chloride concentrations and on ^36^Cl/Cl ratios. Ultra-pure reagents were used to minimize contamination with natural chloride. Chloride was extracted by precipitation of purified AgCl. Precipitates were dried and pressed in 6.6 mm diameter Ni-target holders. The measured ^36^Cl/Cl of spiked samples are at least one order of magnitude above spiked blanks (^36^Cl/Cl = 4.0 10^−15^ at at^−1^, n = 12) and the calculated ^36^Cl/Cl sample ratios were corrected from the blanks. Measured ^36^Cl/Cl of unspiked samples are at least one order of magnitude above unspiked blanks (^36^Cl/Cl = 1.0 10^−15^ at at^−1^, n = 19). Total uncertainties, including internal errors and external reproducibility, on the determination of ^36^Cl/Cl ratios and chloride concentrations by ID-AMS were respectively estimated at 7% and 5%^50^.

The calculations made on the ^36^Cl data are succinctly described in the main paper, and more extensively in the Supplementary Materials.

## Supplementary information

Supplementary Information
