# Supplementary material for: Water transit time and active recharge in the Sahel inferred by bomb-produced 36Cl
Source: Sci Rep. 2019 May 16;9:7465. doi: 10.1038/s41598-019-43514-x (PMC6522497; doi:10.1038/s41598-019-43514-x)
Supplement: Supplementary file 1 — Supplementary Information [file 41598_2019_43514_MOESM1_ESM.docx]

Supplementary Materials for

Water transit time and active recharge in the Sahel inferred by bomb-produced ^36^Cl

Camille Bouchez^1,2 *^, Pierre Deschamps^1^, Julio Goncalves^1^, Bruno Hamelin^1^, Abdallah Mahamat Nour^1,3^, Christine Vallet-Coulomb^1^, Florence Sylvestre^1^

^1^ Aix Marseille Univ, CNRS, IRD, INRA, Coll France, CEREGE, Aix en Provence, France

^2^ Univ Rennes, CNRS, Géosciences Rennes, UMR 6118, 35000 Rennes, France

^3^ University of N’Djamena, Département de Géologie, Ndjamena, Chad

*Correspondence to: Camille.bouchez@univ-rennes1.fr

1. Study site description

Lake Chad Basin (LCB) is a 2.5 million km^2^ hydrological closed drainage basin located in central Africa extending between 7° and 24° East and between 5° and 25°North (Fig.1). The northern part of the basin is in Saharian and Sahelian climatic zones, characterized by low rainfall rates (between 0 and 300 mm yr^-1^) and a high evapotranspiration (between 6000 and 2000 mm yr^-1^). The southern Sudanese part of the basin (600,000 km^2^) is the only present-day hydrologically active region of the basin. Rainfall is controlled by the African monsoon, related to the seasonal migration of the Inter Tropical Convergence Zone.

Lake Chad is a shallow endorheic lake with an average water depth of 3 m and a surface of 14,000 km^2^ in April 2013. Lake Chad waters are subject to high evaporation rates of about 2000 mm yr^-1^ with no runoff outlet, yielding a 1 to 2 year residence time of its waters ^1,2^. The spectacular decrease in its surface from 25,000 km^2^ in 1960 to 4,000 km^2^ in 1980 was a consequence of the severe drought that affected the Sahel in the 1980s ^3,4^. Lake Chad surface oscillations thus amplify climatic variations that occur over the hydrological catchment, making Lake Chad an ideal recorder of (paleo)climatic changes.

Modern Lake Chad can be divided into three geomorphological entities: the Northern pool and the Southern Pool are separated by the Great Barrier and the Archipelagos correspond to the extension of the Kanem erg into the Southern Pool (Fig.1)^5^. The three pools either form one lake or interconnected individual sub-lakes depending on the lake level. Different Lake Chad stages have thus been described depending on the lake level ^5,6^. When the lake level exceeds the Bahr El Ghazal sill (282.3m), Lake Chad waters overflow through the presently dried Bahr El Ghazal River and connect the southern sub-basin with the northern sub-basin. The last significant overflows in the Bahr El Ghazal River occurred at the end of the 19^th^ century ^5,7^. The filling of the Northern sub-catchment during humid periods led to the formation of a MegaLake Chad of 350,000 km^2^, whose paleo-geomorphic features were revealed by remote sensing studies ^8,9^. The last MegaLake Chad stage occurred during the mid-Holocene African Humid Period ^10^.

The Chari-Logone River flows from the Central African Republic into the southern shore of Lake Chad and accounts for 90% of the water inputs to the lake ^2^. The Komadougou-Yobe River flows from Nigeria and Niger to the western shore of Lake Chad and provides a minor contribution similar to direct input from precipitations (5%). Because of climatic and topographic South-North gradients, the Chari-Logone drainage basin can be divided into two areas with contrasted water dynamics, the upper basin and the lower plains (Fig. 1)^11^. Significant runoff and weathering occur in the southern upper basin with rapid runoff, loading suspended matter during the rainy season, and a continuous baseflow supply from groundwaters located in alterite rocks ^11^. While the flood peak occurs in October at the outlet of the upper basin, it reaches Lake Chad only in late November (600km away). The lower basin floodplains slow down flood propagation, leading to the one-month transfer time between the upper and lower basins (section 2.3, Fig.E). From a chemical point of view, the lower plains are an evaporative area with a concentration factor of about 3 and deposition of clay minerals and calcite occur in the plains ^11^. Therefore, stream flows, total dissolved salts and suspended materials that reach Lake Chad originate from the upper tropical basin.

The lake and the lower Chari-Logone River are connected to an unconfined aquifer of 500,000 km^2^ in the central part of the LCB, the Quaternary Phreatic Aquifer (QPA) (Fig.1, Fig. A)^12^. This extensive reservoir is the primary source of human water use. The aquifer is composed by silts, sands and clays deposited during alternating extended and restricted lacustrine phases in the Quaternary. The thickness of the aquifer ranges from 0 to 180 m with an average of around 100 m^12^. The scatter in the chemical signature of groundwater demonstrates that it is strongly heterogeneous ^13–15^ and suggests that it may be multi-layered ^16^. The water table is characterized by three major piezometric depressions around the lake (Chari Baguirmi, Kadzell and one located north of the lake)^17^ and two piezometric domes (Harr, Kanem) shown on Fig. 1. Similar features have already been described in other phreatic aquifers of the Sahelian belt ^17,18^. Although they are well characterized, the explanation for such depressions is still debated ^19^. The main assumption remains a high and localized evapotranspiration rate associated with low hydraulic conductivities ^20^. The plausibility of this assumption was confirmed in the LCB by the steady state regional flow model ^21^. In present-day conditions, the aquifer is recharged by diffuse rainfall with a median recharge estimated in Northern Nigeria of around 43 mm yr^-1 22^ and 3.5 mm yr^-1^ estimated in Niger ^23^. Isotopic studies suggest that the main recharge occurred in past humid periods before 4,000 years BP ^23^ and could be related to the mid-Holocene MegaLake Chad episode that covered a large part of the QPA. Seepage from Lake Chad to the QPA is negligible in terms of volume but ensures the chemical regulation of the lake and allows freshwaters to persist despite the evaporative conditions ^2^.

This QPA is separated from the underlying sedimentary aquifers of the Continental Terminal and Pliocene by a thick clay layer ^12^ (Fig.A). These two continental sediment layers, henceforth referred to as Deep Aquifers (DA), are separated aquifers in the Nigerian part and merge below Lake Chad to form a single aquifer with a total thickness exceeding 275 m. They are confined and of artesian type ^12^. DA groundwaters are only exploited in Northern Nigeria.

2. Hydrological and geochemical data

## 2.1 Sampling

Between 2008 and 2015, we collected 13 samples of the Chari-Logone River (Upper and Lower Basins, Table 1), 10 Lake Chad water samples (Southern Pool and Archipelagos, Table 1), 5 rainfall samples, 70 groundwater samples in the QPA and 33 DA groundwater samples for ^36^Cl analysis (Fig.1, Table1). Sampling locations are displayed on Figure 1. Between 2013 and 2014, the Chari-Logone River was sampled monthly down the confluence in Ndjamena, and analysed for chloride concentrations and δ^18^O and δ^2^H.

The Quaternary aquifer (QPA) was sampled within its Chadian south-eastern part and samples were collected from the most important features of the aquifer: in the vicinity of the rivers, of the lake and of the piezometric depressions. Only the hydrogeological domes of the Kanem are missing. QPA water was sampled from hand-pumped bores, the total depths of which are reported in Table 1.

Groundwaters of the deep aquifers (DA) were mainly sampled in Nigeria and Niger as very few boreholes reach the deep aquifers in Chad. DA water was sampled from artesian bores and total depths are provided in Table 1 when available. Screened intervals are restricted to the CT formation (between 200 and 600 m deep).

For accessibility reasons, field campaigns were mostly carried out each year in November-December, which corresponds to the end of the rainy season. However, a few samples were also collected during the dry season (November to April). For security reasons, the Northern Pool was not sampled but we collected water samples from the other two pools, the Southern Pool and the Archipelagos.

On site analysis included temperature, electrical conductivity, dissolved oxygen concentration and pH. Groundwater samples were collected from existing boreholes that had been preliminarily flushed out. Water samples were collected in pre-cleaned HDPE bottles for major ions and ^36^Cl analyses and in pre-cleaned amber glass bottles for stable isotopes and ^14^C analyses. All samples were stored at 4°C.

## 2.2 Analyses

Major ion (Na^+^, K^+^, Ca^2+^, Mg^2+^, Cl^-^, SO4^2-^, NO^3-^) analyses were carried out on filtered aliquots by an ion chromatography technique (IC) at HydroSciences Montpellier and at UPSud-Orsay, with an uncertainty of 5%. The concentrations of bicarbonate ion were determined by titration on site. The charge balance is reported in Table 1.

Based on a statistical classification of QPA samples (section 5.1), four samples with SO_4_-Cl-Na highly concentrated waters were identified and likely indicative of anthropogenic pollution. In order to exclude contaminated samples a threshold value of NO_3_^-^ is often considered ^24,25^. In this evaporative environment, a concentration threshold would not be relevant, and we therefore applied a proportion threshold. Samples with chloride and nitrate together accounting for more than 20% of the sum of anions were also excluded. This threshold value is consistent with previous identification of groundwater contaminated samples ^26^. Therefore, 8 out of 71 groundwater samples were excluded. A diffuse anthropogenic nitrate pollution is unlikely in the Sahel, because there are no developed industries and agricultural practices are extensive and traditional. These eight samples are likely to be due to human and animal waste or cleaning products, infiltrated through improperly sealed domestic wells.

Measurements of ^36^Cl were carried out by Accelerator Mass Spectrometry ^27^ at the French AMS National facility, ASTER at CEREGE ^28^. Because of low chloride contents in surface and some groundwater samples, the addition of a ^35^Cl enriched spike was required to reach a total chloride amount of 2 mg and to precisely determine the Cl concentration using the ID-AMS technique ^29^. Water samples were processed in batches of 10-15 samples with a spiked blank and an unspiked blank at the end of each batch to estimate chemical contaminations on chloride concentrations and on ^36^Cl/Cl ratios. Ultra-pure reagents were used to minimize contamination with natural chloride. Chloride was extracted by precipitation of AgCl, which was then re-dissolved in aqueous NH_3_. A saturated solution of Ba(NO_3_)_2_ was added to allow BaSO_4_ precipitation and removal from the solution by a 0.45 µm mesh filtration. This step is essential due to isobaric interferences of ^36^S with ^36^Cl in AMS measurements. Purified AgCl precipitates were obtained after re-precipitation in nitric solution and washing three times with ultra-pure water. Precipitates were finally dried and pressed in 6.6 mm diameter Ni-target holders. The measured ^36^Cl/Cl of spiked samples was at least one order of magnitude above those of the spiked blanks (^36^Cl/Cl=4.0x10^-15^ at at^-1^, n=12) and the calculated ^36^Cl/Cl sample ratios were corrected from the blanks. The measured ^36^Cl/Cl of unspiked samples was at least one order of magnitude above those of the unspiked blanks (^36^Cl/Cl=1.0x10^-15^ at at^-1^, n=19). Internal errors and external reproducibility on the determination of ^36^Cl/Cl ratios were estimated at 7%. Internal errors and external reproducibility on the determination of the chloride concentration were estimated at 5%^29^.

Carbon isotopic compositions of the Total Dissolved Inorganic Carbon (TDIC) were analysed for 13 quaternary aquifer samples (and 4 deep aquifer samples ^30^).^14^C analyses were conducted at AMS-ARTEMIS, France after extraction of the dissolved HCO_3_^-^ as CO_2_ gas and removal of sulfur when needed. Data and their uncertainties are reported in pMC (Table1).

Water stable isotopic compositions (δ^18^O and δ^2^H) of all surface water and groundwater samples were analysed at CEREGE. Water samples were equilibrated with CO_2_ (10 h at 291 K) and H_2_ (2 h at 291 K with a platinum catalyst) – for δ^18^O and δ^2^H, respectively – in an automated HDO Thermo-Finnigan equilibrating unit and measured on a dual inlet Delta Plus mass spectrometer. All samples were replicated. For technical reasons, some of the samples were analysed for δ^2^H using a Cavity-Ring-Down laser Spectrometer (WS-CRDS, Picarro L1102-i) and also replicated. Oxygen and hydrogen isotope ratios are reported in ‰ relative to the VSMOW-SLAP scale and following the IAEA reference sheet (IAEA, 2009). We used three working standards, previously normalized using the VSMOW2, SLAP2 and GISP international standards. The total uncertainties for the δ^18^O and δ^2^H values were better than 0.1‰ (1σ) and 1‰ (1σ) respectively.

All data are presented in Table S-1.

## 2.3 Chari-Logone streamflows, [Cl^-^] and δ^18^O: literature data

Studies carried out by the ORSTOM, back in the 1960s, provide the most complete dataset of the hydrology and chemistry of the upper Chari and upper Logone Rivers, even though it is important to point out that data were acquired during a more humid period. In this study, we used sodium concentrations and cations + silica concentrations, measured in the Upper Basin between 1968 and 1972 ^11^ (Fig.E). Monthly flows are average values of the years 1956-1974 (Fig.E). During this time period, the annual flow of the Chari-Logone River was 38.10^9^m^3^/yr against 28.10^9^m^3^/yr in the present-day period.

Monthly streamflows after the confluence of the Chari and the Logone Rivers are available between 1952 and 2015 ^4^ (obtained from the “Direction des Ressources en Eau et de la Météorologie”, Ndjamena, Chad). Dissolved major elements and water stable isotopic composition of the Chari-Logone River waters were measured monthly between 2013 and 2014, filling in the lack of precise and reliable chloride concentrations of the Chari-Logone River. Monthly streamflows, chloride concentrations and δ^18^O compositions averaged for the years 2013-2014 are depicted in figure E, together with monthly streamflows averaged over the entire 1952-2015 time period and punctual chloride compositions measured during a recent period (2008-2012) and an older period (1995-1996 ^31^). The 2013-2014 dataset provides continuous data, with low analytical errors, covering the time period investigated in this study. Punctual measurements of chloride concentrations in the Chari-Logone River by AMS between 2010 and 2015 are also consistent with the chloride concentrations measured by IC on monthly samples (Fig.3).

The chloride exported by the Chari-Logone (6.9±3.4 x10^6^ kg yr^-1^) was calculated as the sum of the monthly product of discharge and chloride concentration. The deviation on averaged monthly streamflows was calculated as the standard deviation of monthly streamflows between 1956 and 2015, and accounts for 40%. Although no long-term time series of Cl- concentrations are available, differences between punctual measurements in 1995, 1996, 2013, 2015 and the monthly concentrations measured in 2013 and 2014 are below 10% (Fig. E). This is consistent with a chemostatic behaviour of the catchment where the uncertainty on the total export flux of Cl- is mainly related to the uncertainty on the estimation of discharge^32^. Therefore, the total error on chloride export was evaluated at 50%.

3. Cl and ^36^Cl deposition

## 3.1 Total chloride deposition over the Chari-Logone catchment

The quantification of total (wet+dry) chloride deposition over the Chari-Logone catchment is crucial and the understanding of ^36^Cl relies on the understanding of the chloride cycle. Wet deposition refers to Cl^-^ in rainfall water and dry deposition refers to Cl^-^ directly deposited on the land surface in the form of dust or atmospheric aerosols. Investigations on Cl^-^ depositions at the continental scale in Europe, the USA and Australia show that total chloride deposition is at first order related to the distance from the coast ^33^ (National Atmospheric Deposition Program (NADP)/National Trends Network, 2000). The total chloride deposition is usually acquired from bulk rainfall samplers open all the time. Inland areas in Spain and in the USA show relatively homogeneous deposition with proportions of dry deposition of chloride and chloride-36 between 10 and 30% ^34–36^. This proportion and the total deposition can significantly increase in areas of evaporitic rock outcrops and strong winds ^35^.

Our restricted collection of rainfall was supplemented by measurements made in similar areas around the world, described below, in order to provide the most accurate evaluation of chloride deposition over the Lake Chad Basin. Chloride concentrations vary between 0.06 and 0.16 mg L^-1^ in rainfall waters collected in Ndjamena and in Northern Cameroon (Table 1). Considering a mean annual precipitation of 900 mm/yr over the Chari-Logone catchment, this yields a chloride fallout rate of 0.8+/-0.4 kg/ha/yr. This value is a minimum as it accounts for a point estimation of wet deposition. The IDAF (Atmospheric Chemistry Monitoring Network in Africa) database provides an evaluation of wet deposition across Africa. Precipitation collectors open automatically during precipitation and close when precipitation ceases, providing data on wet fallout. Aerosols are analysed using bulk particle sampling 10m above the ground but probably do not account for dry deposition. Three stations are located in the Sahel (Niger, Mali and Benin) and three stations are located in the humid zone (Benin, Ivory Coast and Cameroon). This database provides long-term weighted chloride depositions with measurements back to the 1990s for most stations. Wet depositions of 3.0 and 3.1 kg/ha/yr have been measured in Ivory Coast and Cameroon ^37^. In the Sahel, respectively at Banizoumbou (Niger), Katibougou (Mali) and Djougou (Benin), wet depositions of 1.82, 1.86 and 2.3 kg/ha/yr have been estimated ^38^. We expect these values, measured in Sahelian zones closer to the coast, to be higher than the wet chloride deposition in the LCB.

The location of the LCB in the centre of Africa and the absence of orographic barriers allow a comparison with fallout rates measured in the centre of the USA or Australia, that vary between 0.5 and 1 kg/ha/yr ^36,39^. As no salty rocks outcrop in the area, the dry deposition rate is likely to be low compared to average estimates (26% of total deposition ^36^). This assumption is also supported by the very low chloride contents of Lake Chad waters and based on it, all previous studies neglected dry deposition in the Lake Chad salinity mass balance ^2^. Therefore, it is clear that chloride fallout rates over Lake Chad are among the lowest in the world. Taking into account both measurements and the literature review, we estimate a total chloride deposition over the Lake Chad Basin of about 1+/-0.2 kg/ha/yr.

## 3.2 Initial ^36^Cl fallout rate calculation

The natural ^36^Cl fallout was calculated by the following equation ^40^: F_36Cl_=RxF_Cl_ where R is the measured ^36^Cl/Cl ratio and F_Cl_ the chloride fallout. A chloride fallout value of 1 +/- 0.2 kg/yr/ha was used (section 3.1). Therefore, the natural ^36^Cl fallout rate deduced from the measured background ^36^Cl/Cl ratio (216±2x10^-15^ at at^-1^) is 9±5 at m^-2^ s^-1^. This value is consistent with the natural ^36^Cl fallout modelling around 10 at m^-2^ s^-1^ between latitudes 10° and 20° in the Northern Hemisphere ^41,42^. The relatively high 216x10^-15^ at at^-1^ value, despite the low fallout rate, is related to the very low chloride content in rainfall that is a consequence of the distance from the ocean (mean of 1100km). This value is consistent with the highest ratio of 228x10^-15^ at at^-1^ measured in Northern and Central Africa in pre bomb groundwaters of the Nubian Aquifer ^43^.

Our widespread measurements of ^36^Cl/Cl ratios in the waters of the Lake Chad Basin show that modern groundwater can provide a very well constrained estimation of the long-term natural ^36^Cl/Cl ratio. This result is of major importance as i) it is the first measurement at this latitude of ^36^Cl fallout with no bomb-effect, ii) it supports latitudinal models and iii) it gives robust information on the initial ^36^Cl/Cl ratio in the area, which is a recognized issue for water age determination using ^36^Cl radioactive decay ^24^.

## 3.3 Determination of averaged ^36^Cl compositions in the Chari-Logone system

The constant ^36^Cl/Cl ratios of waters sampled along the Chari-Logone River show that the chemical and isotopic composition of the Chari-Logone River is inherited from the upper catchment, which is fully consistent with previous studies carried out in the 1960s ^11^ and described in the previous section. The Southern Pool waters have similar compositions to those of the Chari-Logone River in Ndjamena, which is also very consistent with the Southern Pool hydrology fully dominated by Chari-Logone streamflows. Therefore, we merged data coming from the upper Chari-Logone with data from the lower Chari-Logone and the southern Pool, according to sampling time. Data from the upper and lower basins cannot be merged because of the time transfer of about a month between the two catchments (section 2.3). Averaged ^36^Cl/Cl ratios and their uncertainties, calculated according to the mean square weighted deviation (MSWD), are displayed in Table S-3 and Fig.3, as a function of time. These ratios were thereafter used to calibrate the lumped-parameter model and determine water transit time in the Chari-Logone catchment (Fig.3).

## 3.4 Bomb ^36^Cl deposition over Lake Chad Basin between 1950 and 2015

The ^36^Cl/Cl ratio of rainfall between 1950 and 2015 was evaluated for the Chari-Logone catchment using the bomb ^36^Cl deposition simulated by a general circulation model simulation ^44^, divided by the Cl- total deposition estimated for the Chari-Logone catchment (section 3.2).

The ^36^Cl bomb peak has been recorded in ice cores around the world and the most detailed study comes from the Dye-3 core in Greenland ^45,46^. A total ^36^Cl deposition of 2x10^12^ at m^-2^ was calculated at Dye-3, corresponding to a total mass of bomb-produced ^36^Cl of 80kg. Distributed modelling of the ^36^Cl fluxes at the Earth surface was performed between 1952 and 1972 and calibrated based on ^36^Cl measurements performed on eight ice cores worldwide ^44^. This model simulates a 9x10^12^ at m^-2^ total deposition of ^36^Cl over the Chari-Logone catchment, corresponding to a total mass of bomb-produced ^36^Cl of 216kg. Consistently with stronger stratosphere-troposphere exchanges in the subtropics, this value is significantly higher than in the Dye-3 record and is also consistent with a total mass of bomb-produced ^36^Cl of 300kg estimated directly from available information on bomb test characteristics. Most authors used the Dye-3 ^36^Cl record scaled with a latitudinal correction factor ^47^ as input of ^36^Cl deposition to estimate water ages ^48–50^. While the latitudinal factor is useful to discuss natural ^36^Cl deposition, this correction does not take into account the location of nuclear tests that significantly influences the spatial distribution of bomb-produced ^36^Cl. By contrast, this effect is well simulated by the model used here and for instance, discrepancies between the Northern and the Southern hemispheres are reported ^44^. The ^36^Cl bomb peak deposition through time is specific to each study site and its description should be seriously tackled as it directly influences estimates of groundwater residence times. We believe that the simulated ^36^Cl fallout ^44^ is the most robust estimation at a specific location. In this study, we averaged the ^36^Cl bomb-produced simulations at the 9 grid points covering the Chari-Logone catchment to plot the Chari-Logone ^36^Cl deposition between 1952 and 1972. Before 1952 and after 1972, the ^36^Cl deposition rate is set to the natural deposition rate. The potential recycling of the ^36^Cl bomb peak fallout through its storage in the biosphere and its reemission in the atmosphere in the form of CH_3_Cl, while proposed as an explanation for the unexpectedly high ^36^Cl values in the environment ^51,52^, is not taken into account in this simulation. In view of above-described uncertainties, the 1950-2015 ^36^Cl deposition scenario is associated with an arbitrary uncertainty range of +/-20% (Fig.3).

# 4. Extended description of the calculations

## 4.1 Baseflow separation

River discharge can be divided into quickflow, i.e., the water contribution to river flow soon after rainfall and baseflow, i.e., the water with longer residence times in the catchment that sustain riverflows between rainfall events. Multiple sources of water contribute to both quickflow and baseflow components and they can change with different phases of the hydrological cycle as well as with wet or dry years ^53,54^. This study deals with monthly time steps and therefore considers quickflow in terms of young water with a residence time below 2 months (which aggregates direct runoff and hypodermic runoff) and baseflow in terms of water with higher transit times, in accordance with previous studies ^55,56^. Several techniques have arisen to estimate baseflows based on discharge hydrographs from graphical separation techniques to filter techniques but they involve a significant degree of user subjectivity ^53^. These techniques have been developed for daily streamflows and are not fully accurate for monthly hydrographs. The increasing amount of geochemical data measured in rivers and groundwaters has also been used to estimate groundwater inflows to rivers and quantify baseflow. Here, we used a combination of several techniques to estimate baseflow supplying the upper Chari-Logone River as reliably as possible: 1) a digital filter on monthly streamflow, 2) sodium mass balance and 3) isotopic mass balance.

1. Digital Filter

A digital filter^57,58^ was applied to monthly streamflows between 1955 and 2015 :

$$b_{k}=a\times b_{k-1}+\frac{1+a}{2}\times\left( y_{k}-y_{k-1} \right)$$

where b_k_ is the baseflow flux at time k, y_k_ the total discharge at time k and a the recession constant. As this method was initially developed on daily streamflows and thus adapted to temperate climate events of a few days, its ability to provide relevant estimates using monthly streamflows is unclear. Because of the climatic regime of the study site dominated by the monsoon, with one short rain season and a long dry season, the annual hydrograph shows a similar rising limb and shape as an event hydrograph after one rain event in a temperate climate, but at monthly and not daily time scales. Therefore, the hydrology is dominated by a monthly flood and daily flow events are negligible. An attempt to apply this method on monthly streamflow is made here. The digital filter on monthly streamflows using the common value of parameter a of 0.95 yields a mean annual baseflow of 57+/-11% of annual streamflow between 1957 and 2015.

1. Mass Balance

$$b_{k}=y_{k}\times\frac{C_{R}- C_{SW}}{C_{GW}- C_{SW}}$$

where C_R_, C_SW_ and C_GW_ are the chemical compositions of the river, surface runoff and groundwater. This equation assumes that the river composition is only due to the mixing between surface runoff and groundwater, which in our case is only valid in the upper basin as the lower basin is dominated by evaporative processes. In this calculation we assume time invariant groundwater composition. This assumption might yield errors, particularly as it is likely that different parts of the groundwater system could contribute to the baseflow as the rainfall changes.

Chloride concentrations were not available in the upper catchment from literature data. We therefore separated quick flows and groundwater flows using the sum of cations (including silica) concentrations ^11^ and using sodium concentrations. Concentrations of the dry season (November to April) were used as the groundwater end-member ([cations+silica]_GW_=34mg/L; [Na+]_GW_=3.3mg/L; Fig. E). Concentrations of quick flows were estimated assuming that flows during the early rainy season (May-July) result from a mixing of surface runoff waters and a groundwater contribution equal to the baseflow during the dry season, as groundwater levels only increase after several months of rain ([cations+silica]_SW_=24.7mg/L ; [Na^+^]_SW_=1.85mg/L ; Fig. E). An annual proportion of baseflow between 54 and 60% was estimated.

1. Amplitude Ratio using δ^18^O signals in rainfall and streamflow

The proportion of the young water fraction in streamflow, called quickflow in this study, can be estimated by tracer cycles even from heterogeneous catchments ^55^. Following this technique, we calculated the best fit sine coefficients of the δ^18^O cycles of the Chari-Logone River and the precipitation at Ndjamena but also Douala and Kano which are the closest GNIP stations. The fitted amplitudes are 2.8 for the river, 5 at N’Djamena and 4 at Douala and Kano. The amplitude of the River signal is a maximum because it reflects high evapoconcentration in the downstream River and upstream data would have been more relevant. This yields a maximum estimation of 0.55 for the proportion of quickflow. The resulting minimum estimation of 45% of baseflow is lower than previous estimates.

Although considerable limitations are associated with each method due to the restricted amount of data available, baseflow estimates are consistent and yield an annual estimation of baseflow around 0.6+/-0.1, and an annual proportion of solutes brought by baseflow in the river between 0.7+/-0.1.

## 4.2 Lumped parameter models

In the subsurface, water transit through different flowpaths and therefore outflows are mixtures of water with varied transit time. As a result, tracer outputs are different from tracer inputs as they integrate this mixing. The output tracer concentration is related to the input tracer concentration through a convolution integral:

$$C_{out} \left( t \right)= \int_{0}^{\infty} C_{in} \left( t-\tau\right)h(\tau)d\tau$$

where C_in_ and C_out_ are respectively the input and output tracer concentrations in the recharge and in the baseflow. Radioactive decay for ^36^Cl can be neglected as half-life of ^36^Cl is 301 ka. The flow model, h(τ), describes the distribution of water fluxes in the catchment and simple functions are commonly used in the literature and briefly described here.

The piston flow model (PFM) assumes one flow path of one transit time (τ_m_) and a tracer displacement without hydrodynamic dispersion or mixing. The output concentration therefore simply equals the input concentration delayed in time by τ_m_. The PFM can therefore be written as:

$$h(\tau) = \delta(\tau-\tau_{m})$$

where τ_m_(yr) is the single parameter representing the mean transit time. This model can be applied in the case of confined aquifers with a small recharge area^59^.

The exponential model (EM) assumes a vertical stratification of groundwater ages, which increases logarithmically from zero at the water table to ages that approach infinity at the base of the aquifer (Jurgens 2012). The EM is given by :

$$h(\tau) = \frac{1}{\tau_{m}}e^{- \frac{\tau}{\tau_{m}}}$$

where τ_m_(yr) is the single parameter representing the mean transit time. This model typically describes homogeneous, unconfined aquifers of constant thickness receiving uniform recharge.

The exponential piston flow model (EPM) combines exponential flow and piston flow transit times. The EPM is given by:

$$h\left( \tau\right)=0 for \tau<\tau_{m}(1-f)$$

$$h\left( \tau\right)= \frac{1}{f\tau_{m}}e^{- \frac{\tau}{f\tau_{m}}+\frac{1}{f}-1} \mathrm{for}\tau>\tau_{m}(1-f)$$

where f(-) is the fraction of the exponential volume to the total volume and τ_m_(yr) the mean transit time. Thus this model has two parameters. The EPM can describe either an aquifer with an unconfined portion following EM assumptions connected to a confined portion following PFM, or piston flow transport through the unsaturated zone followed by exponential mixing ^59^.

The Dispersion model (DM) assumes a semi-infinite medium and tracer transport controlled by advection and dispersion. The DM is given by :

$$h\left( \tau\right)= \frac{1}{\tau_{m}}{.\frac{1}{{\sqrt{4\pi DP\frac{t}{\tau_{m}}}}} e}^{-\frac{\left( 1-\frac{t}{\tau_{m}} \right)^{2}}{4\pi DP\frac{t}{\tau_{m}}}}$$

where τ_m_(yr) is the mean transit time and DP is the dispersion parameter (the inverse of the Peclet number).

The gamma model represents fractal fluctuations of tracers, and can therefore reproduce the long chemical memory of past inputs. The Gamma model is given by:

$$h(\tau) = \frac{\tau^{\alpha-1}}{\beta^{\alpha}Г(\alpha)}e^{- \frac{\tau}{\beta}}$$

where α(-) and β(yr) are the two parameters, and the mean residence time is given by αβ ^60^.

In this study we used a Markov Chain Monte Carlo (MCMC) procedure to determine the posterior distributions of the model parameters with a Metropolis-Hasting algorithm. Analytical uncertainties on observed ^36^Cl/Cl in the Chari-Logone were taken into account in the calculation of the lognormal likelihood function of the Metropolis-Hasting algorithm. The input function, the ^36^Cl/Cl ratio time series, was calculated from the simulated anthropogenic deposition of ^36^Cl for Lake Chad +/- 20% (section 3.3), Lake Chad chloride fallout between 1+/-0.2 kg/ha/yr (section 3.2) and a baseflow proportion between 0.5 and 0.7 (section 4.2). Ten input functions were chosen within the uniform distribution of the^36^Cl/Cl ratio time series range, and for each input function the MCMC procedure was run (10 000 times, with a global acceptance criterion of 15%). A posteriori distributions of the two parameters (MTT : Mean Transit Time, f : ratio of exponential to piston flow) were calculated based on the accepted set of parameters of all Metropolis-Hasting runs. Therefore, the distributions of parameters reflect the deviation which would still match ^36^Cl/Cl input of rainfall within its deviation, as well as the measured ^36^Cl/Cl ratios within their standard analytical error. The results of the EPM model are presented on Figure 3. Posterior distributions indicate a mean residence time in the aquifer of 9.5 +/-2 years and a proportion of exponential flow of 0.8+/-0.2.

The choice of the flow model is a priori and perceptual of the system. The EPM model best suits a priori our system of an unconfined aquifer with one outlet. However, in order to estimate errors arising from the choice of the model, the calibration procedure was carried out using the Gamma Model as well as the Dispersion Model. The two parameters of the gamma model are not well constrained because they are correlated. However, the mean residence time (α.$\beta$) is better constrained with values between 2 and 12, and a maximum of likelihood of 3 years. The two parameters of the Dispersion Model are also highly correlated, which precludes a constrained estimation of the MTT. Accepted values of MTT range between 2.5 and 12 years with a maximum of likelihood of 3 years. The dispersion coefficient ranges between 0.2 and 1.2. It is evident that the 4 years of observed data, although showing a clear decreasing trend, do not constrain the model enough to solve the equifinality issue raised about the two parameters of these two models. To solve this issue, other tracers would have been needed. The estimate of MTT using the EPM falls within the high range of MTT determined by the other models, but could be overestimated.

## 4.3 Hydrologically active surface area calculation

The annual delivery of Cl to the upper Lake Chad Basin (S=330 000 km^2^, 55% of the total surface of the Lake Chad Basin, 600 000 km^2^) was estimated to be 3.3x10^7^ kg/yr, based on a total chloride fallout estimate of 1+/-0.2 kg/ha/r (section 3.2). Annual quantities of Cl transported out of the basin can be estimated from river flows and Cl concentrations. Cl time series during the years 2013-2014 (section 2.3) and DREM flow data yield an annual Cl riverine transport by the Chari-Logone of 6.9x10^6^ kg/yr. The chloride balance in the Chari-Logone catchment is clearly unbalanced and chloride inputs greatly exceed chloride outputs. If climate is considered as the first determiner of the connection or disconnection of an area, the 12% of connected area would therefore correspond to the 12% most humid area. With average annual precipitation (P) and evapotranspiration (PET) rates of 1350mm/y and 1400mm/y in this area, we then calculated a P/PET threshold for connection of 0.95. This value is a maximum and does not take into account soils, seasonality or spatial distribution and distance from the river.

## 4.4 Binary mixing model and calculation of recharge in the Quaternary Aquifer

Fig. Fa shows the ^36^Cl/Cl ratio as a function of Cl^-^ concentration measured in groundwater of the Quaternary Aquifer. Two mixing lines are drawn with two present-day endmembers to take into account the variability of the ^36^Cl/Cl ratio of surface water over the last 50 years. The two surface water endmembers have Cl- concentrations of 0.1 mg/L and ^36^Cl/Cl ratios of 1500 and 20000x10^-15^at/at. The old groundwater endmember has a Cl- concentration of 5 mg/L and a ^36^Cl/Cl ratio of 250 x10^-15^at/at. In this diagram, evaporation is represented by horizontal arrows as it increases Cl^-^ concentration without changing the ^36^Cl/Cl ratio.

Two old groundwaters have a ^36^Cl/Cl ratio below the natural background, probably related to the dissolution of halite and thus cannot be explained by the binary mixing model. Three groundwater samples are below the two mixing lines, and thus cannot be interpreted as an evaporated mixing of recent and old groundwaters, but are very likely related to diffuse recharge by rainfall. The proportion of recent groundwater and evaporation rates for all other groundwater samples was calculated as follows:

$$x=\frac{{Cl}_{O-GW}\left( {\frac{{}^{36}Cl}{Cl}}_{O-GW}-{\frac{{}^{36}Cl}{Cl}}_{S} \right)}{{Cl}_{M-GW}\left( {\frac{{}^{36}Cl}{Cl}}_{S}-{\frac{{}^{36}Cl}{Cl}}_{M-GW} \right)+ {Cl}_{O-GW}\left( {\frac{{}^{36}Cl}{Cl}}_{O-GW}-{\frac{{}^{36}Cl}{Cl}}_{S} \right)}$$

$$E=1-\frac{\left( x\times{Cl}_{M-GW}+\left( 1-x \right)\times{Cl}_{O-GW} \right)}{{Cl}_{S}}$$

With S: sample, O-GW: old groundwater end-member, M-GW: surface water or modern groundwater

For each sample, x and E were calculated twice, using the two surface water endmembers. Averaged values with error bars (differences between the two calculations) are displayed on Fig. F. An average proportion of modern groundwater of 58% and an average evaporation rate of 81% were calculated for all samples. However, because of the heterogeneity of groundwater recharge in the area, we calculated these values per group: close to the hydrological network (group 1), modern groundwaters account for 94±8% with an evaporation of 71±20%; far from the hydrological network (group 3), modern groundwaters account for 19±32% and evaporation for 85±19% ; and in the intermediate group, modern groundwaters account for 61±41% and evaporation for 68±25%. The ^36^Cl distribution in groundwaters also depends on the depth of the boreholes (Fig. G). A depth of 40m significantly displays populations of boreholes enriched or not enriched in ^36^Cl (p-value=0.016), showing that present day recharge is limited to the upper 40m, corresponding to half of the total thickness of the aquifer. The renewal rate can be calculated as the volume of modern water divided by the volume of the aquifer and divided by the time since this water was recharged. In this study, the time of recharge is marked out by nuclear tests, which started in 1952. The renewable rate is thus equal to 0.94*0.5/(2015-1952) = 0.74±0.06 % yr^-1^ and 0.19*0.5/(2015-1952) = 0.1±0.3% yr^-1^ respectively for groundwater close to and far from the hydrological network. These values are in the range of recharge renewal rates calculated for Sahelian aquifers ^23,61^. It corresponds respectively to recharge rates of 78±7 mm yr^-1^ and 16±27 mm yr^-1^, using values of the mean saturated thickness and porosity of the aquifer in the study area (50x10^3^ km^2^) of 35m and 0.3. These values are consistent with independent estimations of recharge in the southern part of the Lake Chad Basin ^21,62^ as well as global estimates of renewable resources ^63^. The volume of the QPA was estimated to be between 5-15x10^12^ m^3 1^. As the studied area (1/10 of all the QPA) corresponds to the recharge area of the aquifer, our calculations indicate a total residence time of the water in the QPA of about 3000-9000 years, which is consistent with Holocene waters found in the centre of the depressions. For comparison, the Chari-Logone flows vary between 20 and 40 km^3^/year, and the present-day withdrawal from the QPA has been estimated to be around 110 hm^3^/yr ^63^, thus around ten times less than recharge water.

5. Extended description of the geochemical signatures of groundwaters of the QPA

## 5.1 Major ion compositions

The chemical facies of all water samples are shown on a Piper diagram (Table 1, Fig.B). The conductivities are displayed by the size of the circles. Surface waters are HCO_3_-Ca type and deep aquifer groundwater is Cl-SO_4_-Na type. Hierarchical groups of QPA groundwater samples were obtained from a Ward classification based on major element concentrations (Na^+^, K^+^, Ca^2+^, Mg^2+^, Cl^-^, SO_4_^2-^, NO_3_^-^), normalized by the conductivity to exclude evaporative processes. The first group consists of low concentrated, HCO_3_-Ca type water samples, chemically similar to surface waters. The second group is made of Na-enriched, moderately concentrated samples. The third group comprises SO_4_-Cl-Na highly concentrated waters. Samples of the fourth group contain high concentrations of nitrate and chloride, representative of anthropogenic pollution. The means and standard deviations of major element concentrations in water samples are shown in Table 2 for each group. We observe an evolution of the QPA groundwater chemistry from an HCO_3_-Ca type to a Cl-SO_4_-Na type together with a significant increase in EC.

It is very interesting to observe a broad consistency between the ^36^Cl/Cl ratio and the chemical composition of groundwaters. Groups 1 and 2 are characterized by enriched ^36^Cl contents while groups 3 and 4 are characterized by low ^36^Cl contents (Table 2). Consistently, pre-modern groundwater shows more concentrated and sodic waters than modern groundwater, highlighting that higher water residence times favour water-rock interaction and Ca/Na exchanges.

## 5.2 Water stable isotopic compositions

The measured δ^18^O and δ^2^H of surface waters are consistent with previous studies of rainfall and surface water isotopic composition ^64,65^ (Fig.C). The isotopic data of rainfall in Ndjamena^64^ define the local meteoric water line δ^2^H=6.3xδ^18^O+4.3 with mean weighted averages of δ^18^O=-3.9‰ and δ^2^H=-21.3 ‰. The δ^18^O values of the downstream Chari-Logone River vary between -6‰ and +3‰ and the Lake Chad δ^18^O values vary between -1‰ and +10‰ ^2,65^. The Chari-Logone River and Lake Chad waters plot along an evaporation line with an evaporative slope of 5‰, revealing the well described evaporative system of the Lake Chad Basin ^2,65^. The deep aquifer groundwaters show completely different isotopic compositions with δ^18^O varying between -5‰ and -7.1‰ and all points plot below the evaporation line of the present day water cycle (Fig.C). This supports the disconnection of this aquifer from present-day surface waters.

The δ^18^O compositions of the QPA groundwaters show a large range of variation between -6‰ and +8‰ (Fig.C). QPA waters are divided between samples with isotopic compositions aligning with surface water samples, and samples that plot below the mean weighted present-day surface water compositions. The isotopic compositions suggest that the latest QPA groundwaters cannot have been recharged by present-day waters. One sample out of all QPA samples has water isotopic compositions similar to the DA samples and together with the fact that this sample has the lowest ^36^Cl/Cl ratio measured in the QPA (^36^Cl/Cl=104x10^-15^ at/at), we suggest that this QPA sample could be a DA sample.

Stable isotopic compositions of groundwater of the QPA thus suggest different recharge dynamics (either related to present-day surface waters or not) which is even more relevant when plotting ^36^Cl/Cl as a function of δ^18^O (Fig. D-a). In this plot, present-day recharge end-members (Rainfall, Chari-Logone and Lake Chad) are depicted based on their measured δ^18^O and ^36^Cl/Cl compositions. The expected pre-1950 Lake Chad is also depicted assuming a similar δ^18^O composition range as nowadays and a ^36^Cl/Cl ratio equal to the natural level (216x10^-15^ at at^-1^). Most QPA samples plot within the framework described by these end-members and can be interpreted as either recharged by present-day rainfall, Chari-Logone, Lake Chad or pre-1950 Lake Chad. The geographic position of each sample is highly consistent with the origin of recharge suggested by the ^36^Cl and δ^18^O plot (Fig. D-b). Here, we validate the use of ^36^Cl together with δ^18^O to trace the origin and time of recharge in a Sahelian phreatic aquifer. We observe that only two samples have isotopic signatures consistent with diffuse recharge by rainfall in the system. Some samples (red rectangle) plot outside the designed end-member framework and show homogeneous isotopic composition with ^36^Cl/Cl around 200x10^-15^ at/at, δ^18^O between -4‰ and -2‰ and high conductivities (Fig. D-b). These samples plot away from the present-day evaporation line in the δ^18^O-δ^2^H plot. Moreover, these samples all belong to the chemical group 3 corresponding to the most evolved groundwater. All this evidence suggests old groundwaters, probably recharged during past humid periods as shown by the depleted mean isotopic compositions. Their ^36^Cl/Cl ratio is similar to the present-day natural level, which suggests Holocene waters, as Pleistocene ^36^Cl/Cl ratios are expected to be lower ^66^. These groundwater samples are located in and around the piezometric depressions of the Quaternary Aquifer. Our results are consistent with the old assumption of Mega-Lake waters trapped in the piezometric depression ^67^.

## 5.3 Total Dissolved Inorganic Carbon isotopic compositions

Total dissolved inorganic carbon (TDIC) analyses show ^14^C ratios all over 50 pMC, going from 54 to 92 pMC. Except for two samples with a δ^13^C lower than -20‰, δ^13^C range between -10‰ and -4‰, indicating different sources of the TDIC. Dissolution of carbonates is likely to explain the shift of δ^13^C towards 0‰. However, most samples lie above the mixing line between equilibrated soil gas and carbonates, which indicates an open system and a reequilibration with atmospheric ^14^C. These data cannot be derived into groundwater ages, as they trace other processes than ^14^C decay, which is also demonstrated by inconsistent results from the different ^14^C age models ^68–74^.. This supports the well-known limitations of ^14^C as a groundwater age tracer when considering groundwaters recharged over the last millennia, whose TDIC signature has been mainly acquired within the “partially-open” unsaturated zone^75^. Nevertheless, ^14^C data point to Holocene recent groundwaters with a maximum age of a few millennia, which is fully consistent with ^36^Cl-based interpretations. This illustrates the complexity of ^14^C in such hydrogeological settings and the need for other groundwater age tracers.

# References

1. Roche, M.-A. Traçage naturel salin et isotopique des eaux du système hydrologique du lac Tchad. (ORSTOM, 1980).

2. Bouchez, C. *et al.* Hydrological, chemical, and isotopic budgets of Lake Chad: a quantitative assessment of evaporation, transpiration and infiltration fluxes. *Hydrol Earth Syst Sci* **20**, 1599–1619 (2016).

3. Lemoalle, J., Bader, J.-C., Leblanc, M. & Sedick, A. Recent changes in Lake Chad: Observations, simulations and management options (1973–2011). *Glob. Planet. Change* **80**–**81**, 247–254 (2012).

4. Bader, J.-C., Lemoalle, J. & Leblanc, M. Modèle hydrologique du Lac Tchad. *Hydrol. Sci. J.* **56**, 411–425 (2011).

5. *Hydrologie du lac Tchad*. (Editions de l’ORSTOM, 1996).

6. Tilho, J. Sur l’ordre de grandeur des variations de profondeur et d’étude du Lac Tchad. *CR Acad Sci* **180**, 1233–1236 (1925).

7. Maley, J. Etudes palynologiques dans le bassin du Tchad et paléoclimatologie de l’Afrique nord-tropicale de 30 000 ans à l’époque actuelle. (ORSTOM, 1981).

8. Leblanc, M. J. *et al.* Evidence for Megalake Chad, north-central Africa, during the late Quaternary from satellite data. *Palaeogeogr. Palaeoclimatol. Palaeoecol.* **230**, 230–242 (2006).

9. Schuster, M. *et al.* Holocene Lake Mega-Chad palaeoshorelines from space. *Quat. Sci. Rev.* **24**, 1821–1827 (2005).

10. Amaral, P. G. C. *et al.* Palynological evidence for gradual vegetation and climate changes during the African Humid Period termination at 13°N from a Mega-Lake Chad sedimentary sequence. *Clim Past* **9**, 223–241 (2013).

11. Gac, J.-Y. Géochimie du bassin du lac Tchad : Bilan de l’altération de l’érosion et de la sédimentation. (ORSTOM, 1980).

12. Schneider, J. L. & Wolff, J.-P. *Carte géologique et cartes hydrogéologiques à 1/1 500 000 de la République du Tchad: mémoire explicatif*. **2**, (Éditions du BRGM, 1992).

13. Zairi, R. Étude géochimique et hydrodynamique du Bassin du Lac Tchad : la nappe phréatique dans les régions du Kadzell (Niger oriental) et du Bornou (Nord-Est du Nigéria). (Montpellier 2, 2008).

14. Gaultier, G. Recharge et paléorecharge d’une nappe libre en milieu sahélien (Niger oriental) : approches géochimique et hydrodynamique. (Université de Paris-Sud. Faculté des Sciences d’Orsay (Essonne), 2004).

15. Abderamane, H., Razack, M. & Vassolo, S. Hydrogeochemical and isotopic characterization of the groundwater in the Chari-Baguirmi depression, Republic of Chad. *Environ. Earth Sci.* **69**, 2337–2350 (2013).

16. Ngatcha, B. N., Mudry, J., Aranyossy, J.-F., Naah, E. & Reynault, J. S. Apport de la géologie, de l’hydrogéologie et des isotopes de l’environnement à la connaissance des “nappes en creux” du Grand Yaéré (Nord Cameroun). *Rev. Sci. EauJournal Water* **20**, 29–43 (2007).

17. Aranyossy, J. F. & Ndiaye, B. Étude et modélisation de la formation des dépressions piézométriques en Afrique sahelienne. *Rev. Sci. Eau* **6**, 81 (1993).

18. Archambault, J. L’alimentation des nappes en Afrique Occidentale. *Cpt R L’Hydro Soc Hydro Fr.* **383**, (1960).

19. Lopez, T. *et al.* Subsurface Hydrology of the Lake Chad Basin from Convection Modelling and Observations. *Surv. Geophys.* **37**, 471–502 (2016).

20. Leblanc, M. Application of Meteosat thermal data to map soil infiltrability in the central part of the Lake Chad basin, Africa. *Geophys. Res. Lett.* **30**, (2003).

21. Boronina, A. & Ramillien, G. Application of AVHRR imagery and GRACE measurements for calculation of actual evapotranspiration over the Quaternary aquifer (Lake Chad basin) and validation of groundwater models. *J. Hydrol.* **348**, 98–109 (2008).

22. Edmunds, W., Fellman, E., Goni, I. & Prudhomme, C. Spatial and temporal distribution of groundwater recharge in northern Nigeria. *Hydrogeol. J.* **10**, 205–215 (2002).

23. Leduc, C., Sabljak, S., Taupin, J.-D., Marlin, C. & Favreau, G. Estimation de la recharge de la nappe quaternaire dans le Nord-Ouest du bassin du lac Tchad (Niger oriental) à partir de mesures isotopiques. *Comptes Rendus Académie Sci. - Ser. IIA - Earth Planet. Sci.* **330**, 355–361 (2000).

24. Davis, S. N., Cecil, D., Zreda, M. & Sharma, P. Chlorine-36 and the initial value problem. *Hydrogeol. J.* **6**, 104–114 (1998).

25. Panno, S. V., Kelly, W. R., Martinsek, A. T. & Hackley, K. C. Estimating Background and Threshold Nitrate Concentrations Using Probability Graphs. *Groundwater* **44**, 697–709 (2006).

26. Kim, R.-H., Lee, J. & Chang, H.-W. Characteristics of organic matter as indicators of pollution from small-scale livestock and nitrate contamination of shallow groundwater in an agricultural area. *Hydrol. Process.* **17**, 2485–2496 (2003).

27. Elmore, D. *et al.* Analysis of 36Cl in environmental water samples using an electrostatic accelerator. *Nature* **277**, 22–25 (1979).

28. Arnold, M. *et al.* The French accelerator mass spectrometry facility ASTER: Improved performance and developments. *Nucl. Instrum. Methods Phys. Res. Sect. B Beam Interact. Mater. At.* **268**, 1954–1959 (2010).

29. Bouchez, C. *et al.* Isotope Dilution-AMS technique for 36Cl and Cl determination in low chlorine content waters. *Chem. Geol.* **404**, 62–70 (2015).

30. Bouchez, C. *et al.* Investigation of 36Cl Distribution: Towards a New Estimation of Groundwater Residence Times in the Confined Aquifer of the LCB? *Procedia Earth Planet. Sci.* **13**, 147–150 (2015).

31. Djoret, D. & Bellion, Y. ETUDE DE LA RECHARGE DE LA NAPPE DU CHARI BAGUIRMI (TCHAD) PAR LES METHODES CHIMIQUES ET ISOTOPIQUES. (Avignon et des pays de vaucluse, 2000).

32. Godsey, S. E., Kirchner, J. W. & Clow, D. W. Concentration–discharge relationships reflect chemostatic characteristics of US catchments. *Hydrol. Process.* **23**, 1844–1864 (2009).

33. Guan, H., Love, A. J., Simmons, C. T., Makhnin, O. & Kayaalp, A. S. Factors influencing chloride deposition in a coastal hilly area and application to chloride deposition mapping. *Hydrol Earth Syst Sci* **14**, 801–813 (2010).

34. Hainsworth, L. J., Mignerey, A. C., Helz, G. R., Sharma, P. & Kubik, P. W. Modern chlorine-36 deposition in southern Maryland, U.S.A. *Nucl. Instrum. Methods Phys. Res. Sect. B Beam Interact. Mater. At.* **92**, 345–349 (1994).

35. Alcalá, F. J. & Custodio, E. Atmospheric chloride deposition in continental Spain. *Hydrol. Process.* **22**, 3636–3650 (2008).

36. Moysey, S., Davis, S. N., Zreda, M. & Cecil, L. D. The distribution of meteoric 36Cl/Cl in the United States: a comparison of models. *Hydrogeol. J.* **11**, 615–627 (2003).

37. Galy-Lacaux, C., Laouali, D., Descroix, L., Gobron, N. & Liousse, C. Long term precipitation chemistry and wet deposition in a remote dry savanna site in Africa (Niger). *Atmospheric Chem. Phys.* **9**, 1579–1595 (2009).

38. Laouali, D. *et al.* Long term monitoring of the chemical composition of precipitation and wet deposition fluxes over three Sahelian savannas. *Atmos. Environ.* **50**, 314–327 (2012).

39. Keywood, M. D., Chivas, A. R., Fifield, L. K., Cresswell, R. G. & Ayers, G. P. The accession of chloride to the western half of the Australian continent. *Soil Res.* **35**, 1177–1190 (1997).

40. Andrews, J. N. *et al.* Chlorine-36 in groundwater as a palaeoclimatic indicator: the East Midlands Triassic sandstone aquifer (UK). *Earth Planet. Sci. Lett.* **122**, 159–171 (1994).

41. Bentley, H. W., Phillips, F. M. & Davis, S. N. Chlorine-36 in the terrestrial environment. *Handb. Environ. Isot. Geochem.* **2**, 427–480 (1986).

42. Blinov, A. *et al.* An excess of 36Cl in modern atmospheric precipitation. *Nucl. Instrum. Methods Phys. Res. Sect. B Beam Interact. Mater. At.* **172**, 537–544 (2000).

43. Patterson, L. J. *et al.* Cosmogenic, radiogenic, and stable isotopic constraints on groundwater residence time in the Nubian Aquifer, Western Desert of Egypt. *Geochem. Geophys. Geosystems* **6**, Q01005 (2005).

44. Heikkilä, U. *et al.* 36Cl bomb peak: comparison of modeled and measured data. *Atmos Chem Phys* **9**, 4145–4156 (2009).

45. Elmore, D. *et al.* 36Cl bomb pulse measured in a shallow ice core from Dye 3, Greenland. *Nature* **300**, 735–737 (1982).

46. Synal, H.-A., Beer, J., Bonani, G., Suter, M. & Wölfli, W. Atmospheric transport of bomb-produced 36Cl. *Nucl. Instrum. Methods Phys. Res. Sect. B Beam Interact. Mater. At.* **52**, 483–488 (1990).

47. Bentley, H. W. *et al.* Thermonuclear 36Cl pulse in natural water. *Nature* **300**, 737–740 (1982).

48. Alvarado, J. A. C. *et al.* 36Cl in modern groundwater dated by a multi-tracer approach (3H/3He, SF6, CFC-12 and 85Kr): a case study in quaternary sand aquifers in the Odense Pilot River Basin, Denmark. *Appl. Geochem.* **20**, 599–609 (2005).

49. Tosaki, Y. *et al.* Application of 36Cl as a dating tool for modern groundwater. *Nucl. Instrum. Methods Phys. Res. Sect. B Beam Interact. Mater. At.* **259**, 479–485 (2007).

50. Tosaki, Y., Tase, N., Sasa, K., Takahashi, T. & Nagashima, Y. Estimation of Groundwater Residence Time Using the 36Cl Bomb Pulse. *Ground Water* **49**, 891–902 (2011).

51. Milton, G. M. *et al.* Evidence for chlorine recycling—hydrosphere, biosphere, atmosphere—in a forested wet zone on the Canadian Shield. *Appl. Geochem.* **18**, 1027–1042 (2003).

52. Cornett, R. J. *et al.* Is 36Cl from weapons test fallout still cycling in the atmosphere? *Radiocarbon* **38**, 17–18 (2006).

53. Cartwright, I., Gilfedder, B. & Hofmann, H. Contrasts between estimates of baseflow help discern multiple sources of water contributing to rivers. *Hydrol. Earth Syst. Sci.* **18**, 15–30 (2014).

54. McCallum, J. L., Cook, P. G., Brunner, P. & Berhane, D. Solute dynamics during bank storage flows and implications for chemical base flow separation. *Water Resour. Res.* **46**, W07541 (2010).

55. Kirchner, J. W. Aggregation in environmental systems-Part 1: Seasonal tracer cycles quantify young water fractions, but not mean transit times, in spatially heterogeneous catchments. *Hydrol. Earth Syst. Sci.* **20**, 279–297 (2016).

56. Jasechko, S., Kirchner, J. W., Welker, J. M. & McDonnell, J. J. Substantial proportion of global streamflow less than three months old. *Nat. Geosci.* **9**, 126–129 (2016).

57. Lyne, V. Stochastic time-variable rainfall-runoff modelling. in

58. Nathan, R. J. & McMahon, T. A. Evaluation of automated techniques for base flow and recession analyses. *Water Resour. Res.* **26**, 1465–1473 (1990).

59. Jurgens, B. C., Böhlke, J. K. & Eberts, S. M. TracerLPM (Version 1): An Excel® workbook for interpreting groundwater age distributions from environmental tracer data. (2012).

60. Kirchner, J. W., Feng, X. & Neal, C. Fractal stream chemistry and its implications for contaminant transport in catchments. *Nature* **403**, 524 (2000).

61. Le Gal La Salle, C. *et al.* Renewal rate estimation of groundwater based on radioactive tracers (3H, 14C) in an unconfined aquifer in a semi-arid area, Iullemeden Basin, Niger. *J. Hydrol.* **254**, 145–156 (2001).

62. Ngatcha Benjamin, N., Mudry, J.-N. & Reynauld Jean, S. *Groundwater Recharge from Rainfall in the Southern Border of Lake Chad in Cameroon*. **2**, (2007).

63. SDEA. *Schema Directeur de l’Eau et de l’Assainissement 2003- 2020*. (2003).

64. IAEA/WMO. Global Network of Isotopes in Precipitation. The GNIP Database. (2017).

65. Fontes, J. C., Gonfiantini, R., Roche, M.-A., Isotope Hydrology 1970 & Isotope Hydrology 1970. Deutérium et oxygène-18 dans les eaux du lac Tchad. in 387–404 (IAEA, 1970).

66. Blinov, A. *et al.* Ratio of 36Cl/Cl in ground ice of east Siberia and its application for chronometry. *Geochem. Geophys. Geosystems* **10**, Q0AA03 (2009).

67. Djoret, D. & Travi, Y. *Groundwater vulnerability and recharge or palaeorecharge in the Southeastern Chad Basin, Chari Baguirmi aquifer*. (International Atomic Energy Agency (IAEA), 2001).

68. Ingerson, E. & Pearson, F. J. Estimation of age and rate of motion of groundwater by the 14C-method. *Recent Res. Fields Atmosphere Hydrosphere Nucl. Geochem.* 263–283 (1964).

69. Tamers, M. A. Radiocarbon Ages of Groundwater in an Arid Zone Unconfined Aquifer. in *Isotope Techniques in the Hydrologic Cycle* (ed. Stout, G. E.) 143–152 (American Geophysical Union, 1967). doi:10.1029/GM011p0143

70. Mook, W. G. The dissolution-exchange model for dating groundwater with 14 C. in *Interpretation of environmental isotope and hydrochemical data in groundwater hydrology* **213**–**225**, (1976).

71. Evans, G. V., Otlet, R. L., Downing, R. A., Monkhouse, R. A. & Rae, G. Some problems in the interpretation of isotope measurements in United Kingdom aquifers. in *Isotope hydrology 1978* **679**–**708**, (1979).

72. Fontes, J.-C. & Garnier, J.-M. Determination of the initial 14C activity of the total dissolved carbon: A review of the existing models and a new approach. *Water Resour. Res.* **15**, 399–413 (1979).

73. Salem, O., Visser, J. H., Dray, M. & Gonfiantini, R. Groundwater flow patterns in the western Libyan Arab Jamahiriya evaluated from isotopic data. *Groundw. Flow Patterns West. Libyan Arab Jamahiriya Eval. Isot. Data* (1980).

74. Eichinger, L. A contribution to the interpretation of 14 C groundwater ages considering the example of a partially confined sandstone aquifer. *Radiocarbon* **25**, 347–356 (1983).

75. Gillon, M. *et al.* Open to closed system transition traced through the TDIC isotopic signature at the aquifer recharge stage, implications for groundwater 14C dating. *Geochim. Cosmochim. Acta* **73**, 6488–6501 (2009).


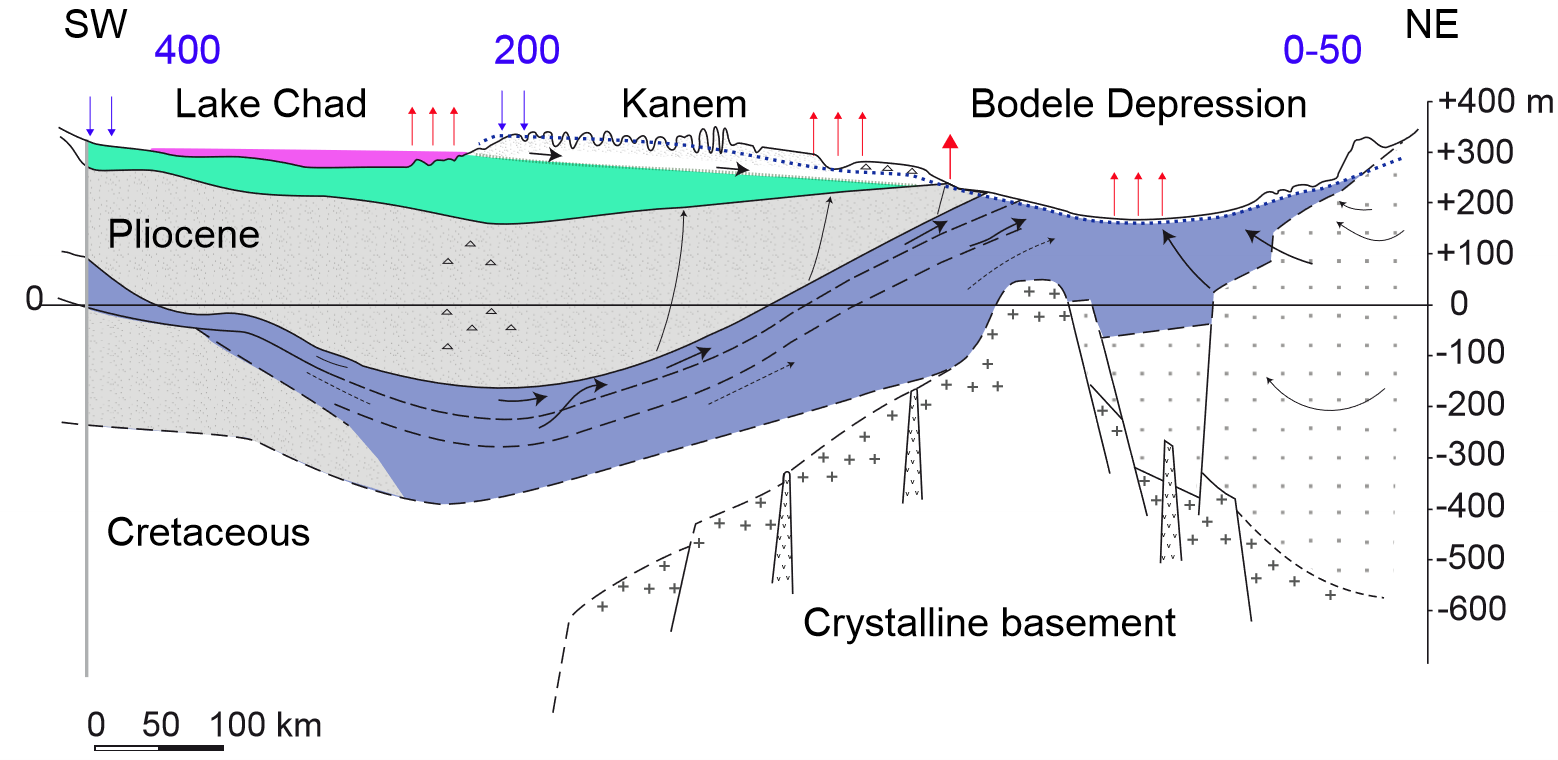


Fig. A. Lake Chad Aquifer System cross-section: Lake Chad (pink), Quaternary Phreatic Aquifer (green), Deep Aquifers (blue).

The piezometric surfaces are drawn by dotted lines, showing the artesian nature of the deep aquifers. Precipitation rates (in mm/yr) are displayed in blue, red arrows show evapotranspiration zones and black arrows show groundwater flow directions (adapted from ^12^)


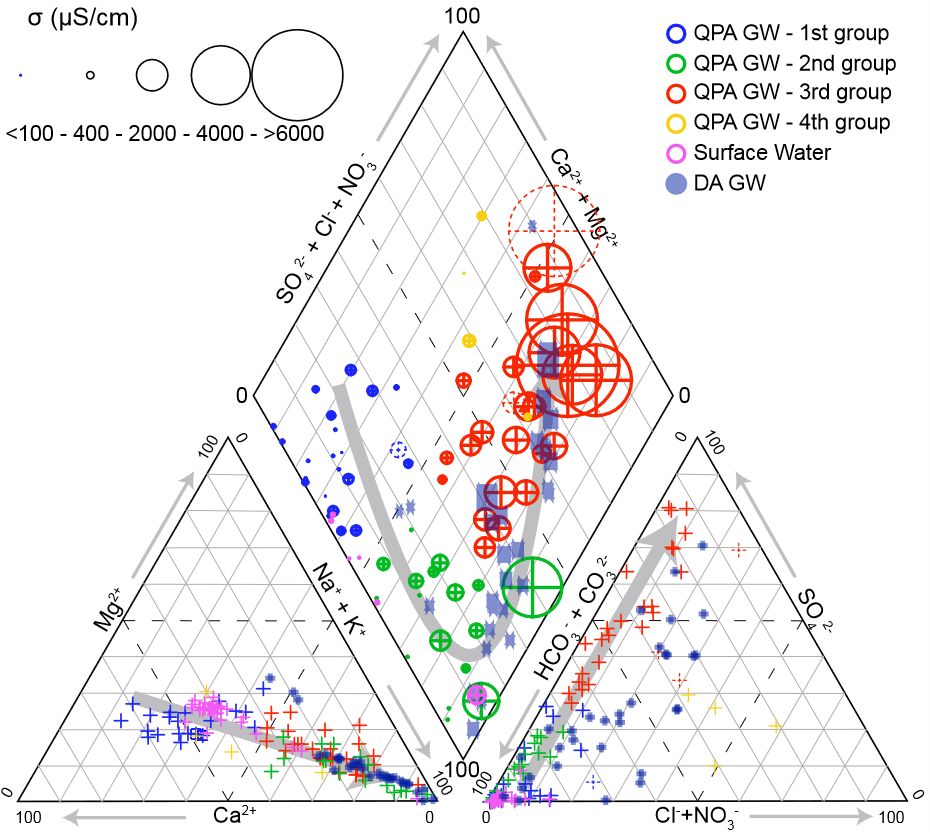


Fig. B. Piper diagram of groundwater samples of the Quaternary Phreatic Aquifer (QPA).

QPA groundwater is depicted with four colours (blue, green, red, yellow) corresponding to the four distinct geochemical groups derived from the cluster analysis. Surface water samples are shown in pink and Deep Aquifer (DA) samples in transparent blue. The size of the circle is proportional to electrical conductivity. Samples from group 3 with a composition of Cl-+NO3- above 20% are plotted by dotted points. These samples and all group 4 samples were excluded from our data analysis due to likely anthropogenic pollution. The piper diagram shows the chemical evolution of groundwater in the QPA with the increasing water residence time.


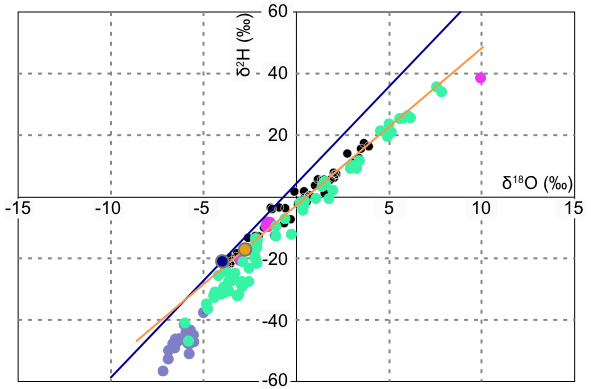


Fig. C. Water isotopic compositions measured in waters of the Lake Chad Basin (Chari-Logone Rivers (black), Lake Chad waters (pink), Quaternary Phreatic Aquifer (green), Deep Aquifers (blue)).

The blue line is the local meteoric water line of Djamena with its long-term weighted average, the orange line is the evaporation line of surface waters with the Chari-Logone long-term weighted average.


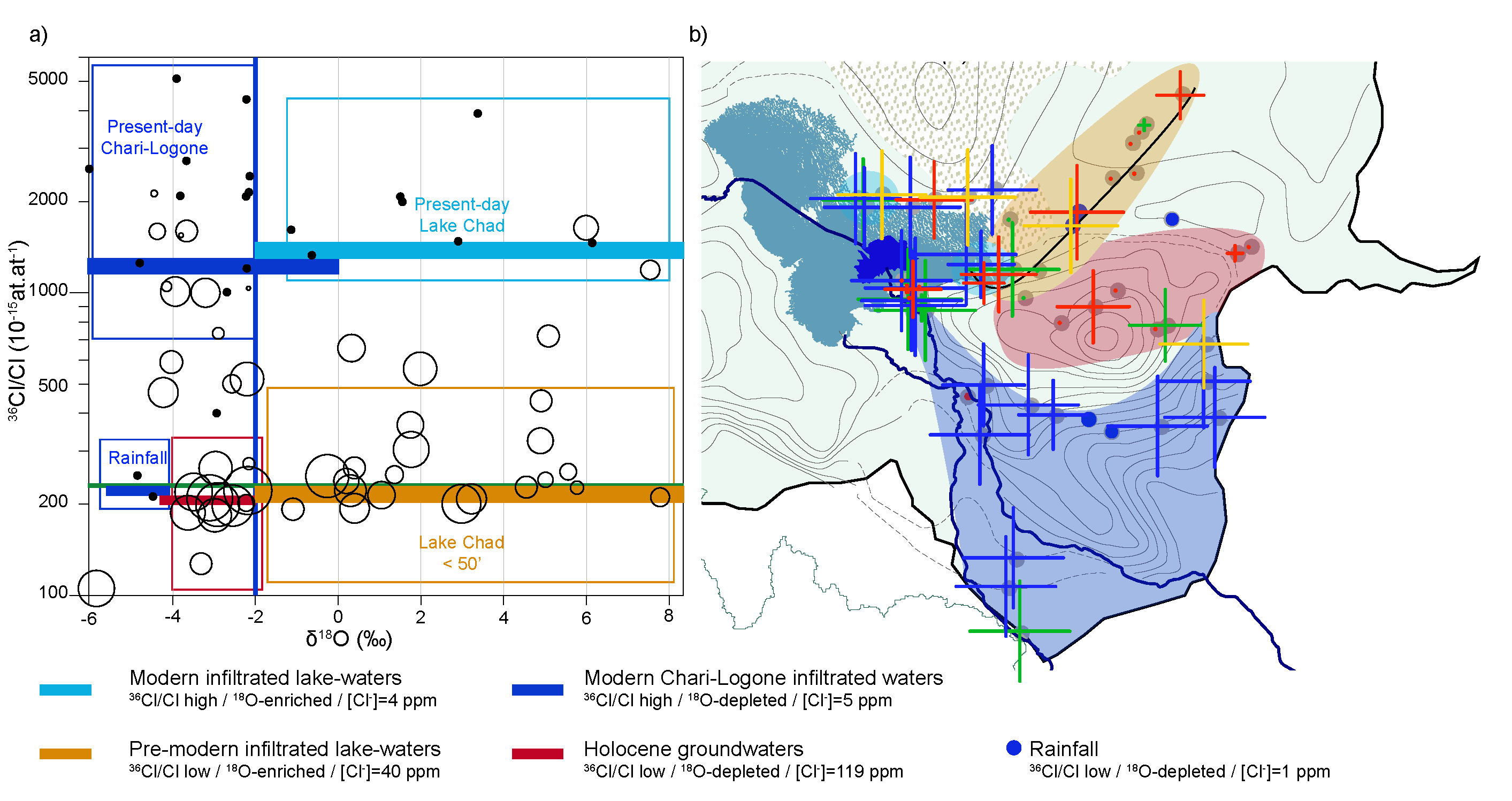


Fig. D. Origin of Quaternary Phreatic Aquifer (QPA) groundwaters, inferred from the good agreement of all geochemical tracers: major elements, stable isotopic compositions and ^36^Cl

1. ^36^Cl/Cl (x10^-15^ at at^-1^) ratios as a function of the δ ^18^O composition (‰). QPA groundwaters are depicted with black circles, size is proportional to the Cl- concentration. ^36^Cl and ^18^O composition ranges of the present day Chari Logone are in dark blue, of the present-day Lake Chad in light blue, of rainfall in dark blue and of Lake Chad before the nuclear tests in orange. In red are values that cannot be explained by these endmembers and that are interpreted as Holocene waters.
2. Location of groundwater masses associated with distinct recharge dynamics highlighted by the ^36^Cl - δ ^18^O cross-plot. Groundwater samples are depicted according to the group they belong to (see Fig. B), and the size of the cross is related to the proportion of modern groundwater (see Fig. F).


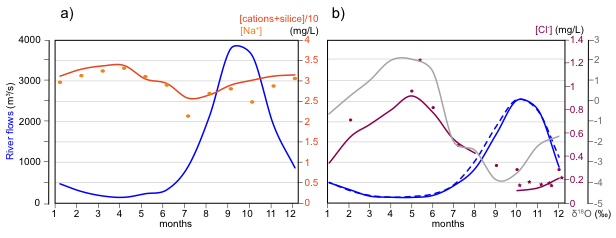


Fig. E. Compilation of hydrological and geochemical data from the literature used in this study to estimate baseflow.

1. Monthly streamflows in the upper catchment, from flow measurements averaged between 1956 and 1974. Averaged sodium concentrations (mg/L) and cations+silica concentrations (10 mg/L) between 1968 and 1972. All data come from ^11^.
2. Monthly streamflows in the lower catchment, from flow measurements averaged between 2013 and 2014 (bold blue line) and between 1956 and 2015 (dotted blue line). Data are taken from the “Direction des Ressources en Eau et de la Météorologie”, Ndjamena, Chad. Chloride concentrations are monthly measurements averaged over 2013-2014 (purple line) and punctual measurements in 1995-1996 (purple dots, ^31^) and punctual measurements in 2010-2015 (purple stars: (20,47)). δ ^18^O compositions are averages of monthly measurements carried out in 2013 and 2014 (grey line).

The comparison between the two hydrographs shows the one-month transfer time of the flood peak between the upper and the lower basins. The apparent reduction in the flows is a bias linked to the time period considered. The chemograph of the downstream catchment shows a higher seasonal amplitude because of intense evaporation.


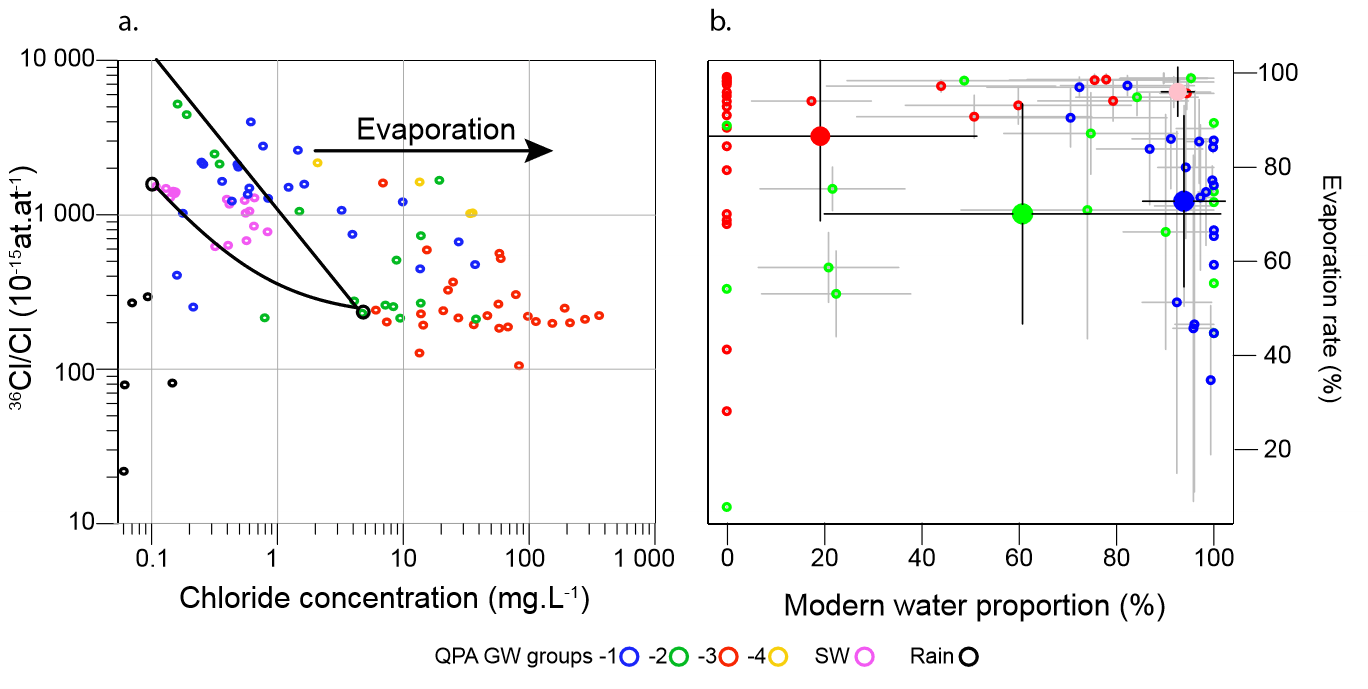


Fig. F. Fraction of modern water, used to quantify recharge of the Quaternary Phreatic Aquifer.

QPA Groundwaters are represented in blue, green, red and yellow, following the clustering classification, surface water samples are in pink and rainfall in black.

1. ^36^Cl/Cl (x10^-15^ at at^-1^) ratios as a function of chloride concentrations. Two mixing lines are drawn, for two surface water endmembers (1: [0.1, 1500] and 2: [0.1, 20000]) and one old groundwater endmember [5, 250]. Evaporation leading to increasing [Cl-] and constant ^36^Cl/Cl ratio is indicated by a horizontal arrow.
2. Proportions of modern water and evaporation rates in QPA groundwaters. These values were calculated from the mixing model in a). Error bars in grey show the uncertainty of the calculation linked to the variability of the ^36^Cl/Cl signature of the two surface water endmembers. Averaged modern water proportion and evaporation rate per group are drawn.


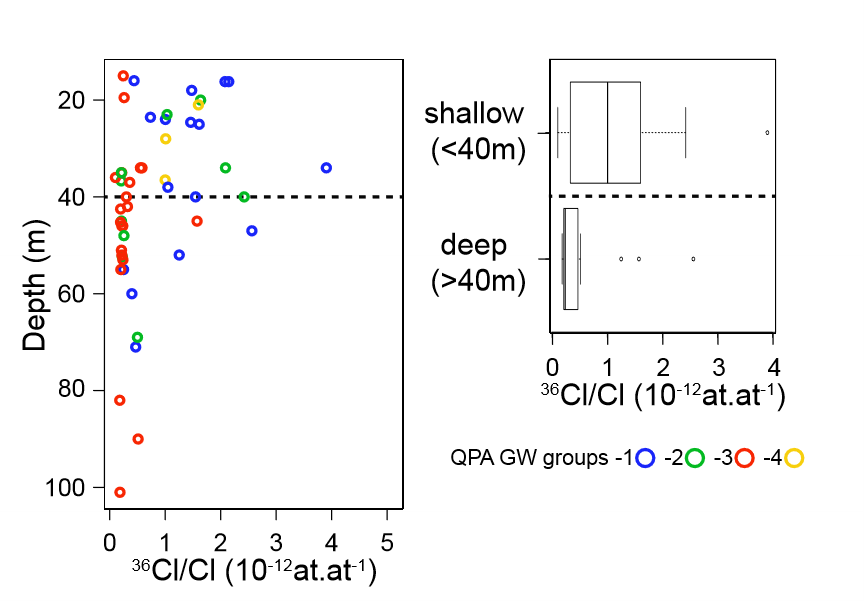


Fig. G. Determination of the depth of renewed groundwaters.

^36^Cl/Cl (x10^-12^ at at^-1^) ratios represented as a function of the depth of the bores. Bore depths were obtained from the “Direction de la Connaissance du Domaine Hydraulique” database and from the “Commission du Bassin du Lac Tchad” database. A Kruskal-Wallis test demonstrated that groundwaters that were sampled below 40 m depth and above 40 m depth are significantly different from each other with respect to ^36^Cl/Cl ratios (p-value = 0.016, chi squared value). Shallow ^36^Cl-enriched groundwaters are therefore interpreted as modern groundwater, whereas ^36^Cl-depleted groundwaters are interpreted as old groundwaters.

Table 1. Raw data acquired in this study. Samples to which a spike was added (*). Samples in italics are samples that were excluded because of potential anthropogenic pollution.

| Sample type | QPA-Group | Date | Lat. °N | Long. °E | EC µS/cm | [Cl^-^] mg/L | ∆ [Cl^-^] mg/L |  | [NO_3_^-^] mg/L | Charge balance % | ^36^Cl/Cl 10^-15^at.at^-1^ | ∆ ^36^Cl/Cl 10^-15^at.at^-1^ | δ^13^C ‰ | ^14^C pMC | ∆ ^14^C pMC | δ^18^O ‰ | δ^2^H ‰ | Total depth m |
| --- | --- | --- | --- | --- | --- | --- | --- | --- | --- | --- | --- | --- | --- | --- | --- | --- | --- | --- |
| GW QPA | 3 | 03/12/2012 | 12,941 | 15,407 | 2390 | 62,84 | 3,66 | * | 2,2 | 3,4 | 554 | 51 | -4,6 | 79,1 | 0,2 | 1,96 | 2,1 | 34 |
| GW QPA | 1 | 28/07/2012 | 12,080 | 17,070 | 133 | 1,77 | 0,10 | * | 7,2 | 8,0 | 1547 | 139 |  |  |  | -3,79 | -30,4 | 40 |
| GW QPA | 1 | 29/07/2012 | 11,681 | 16,301 | 171 | 0,17 | 0,01 | * | 1,5 | 10,5 | 445 | 62 |  |  |  | -2,93 | -27,5 | 58 |
| GW QPA | 2 | 07/08/2012 | 13,384 | 15,487 | 2370 | 40,97 | 0,50 | * | <D.L. | 0,2 | 209 | 10 |  |  |  | 3,23 | 9,2 | 37 |
| GW QPA | 3 | 27/07/2012 | 13,110 | 17,315 | 1660 | 61,76 | 0,50 | * | 29,3 | -2,9 | 260 | 12 |  |  |  | -2,98 | -27,7 | 20 |
| GW QPA | 3 | 27/07/2012 | 13,103 | 17,313 | 5410 | 73,57 | 0,50 | * | <D.L. | -2,3 | 185 | 9 |  |  |  | -3,65 | -30,5 | 101 |
| GW QPA | 2 | 26/07/2012 | 13,377 | 16,785 | 129 | 0,86 | 0,05 | * | 0,5 | 2,4 | 220 | 24 |  |  |  | -4,47 | -32,1 | 46 |
| GW QPA | 3 | 01/12/2012 | 13,709 | 16,314 | 920 | 14,95 | 0,90 | * | 11,9 | 5,3 | 231 | 23 | -4,8 | 70,3 | 0,3 | 4,53 | 21,4 |  |
| GW QPA | 3 | 22/07/2012 | 12,848 | 14,666 | 1158 | 15,59 | 0,50 | * | 1,8 | 1,4 | 191 | 11 |  |  |  | -1,11 | -12,8 |  |
| GW QPA | 1 | 05/08/2012 | 13,548 | 14,274 | 746 | 10,72 | 0,50 | * | 11,5 | 3,4 | 1189 | 55 |  |  |  | 7,55 | 35,6 | 17 |
| GW QPA | 2 | 05/08/2012 | 13,538 | 14,318 | 782 | 14,94 | 0,50 | * | <D.L. | 2,0 | 720 | 34 |  |  |  | 5,09 | 20,9 |  |
| GW QPA | 1 | 29/07/2012 | 11,778 | 16,118 | 85 | 0,23 | 0,03 | * | 0,8 | 9,1 | 284 | 38 |  |  |  | -4,85 | -34,8 | 62 |
| GW QPA | 1 | 08/12/2011 | 12,727 | 14,721 | 318 | 0,63 | 0,03 | * | <D.L. | 0,9 | 1330 | 120 |  |  |  | -0,63 | -7,1 |  |
| GW QPA | 1 | 28/07/2012 | 11,717 | 16,700 | 142 | 0,91 | 0,05 | * | 5,7 | 4,7 | 1262 | 113 |  |  |  | -4,79 | -36,2 | 50 |
| GW QPA | 2 | 01/12/2012 | 12,754 | 15,607 | 938 | 5,11 | 0,29 | * | 0,4 | 5,5 | 236 | 29 | -5,2 | 89,3 | 0,3 | 5,78 | 25,6 | 60 |
| *(GW QPA)* | *3* | *26/07/2012* | *12,680* | *16,170* | *1767* | *64,46* | *0,50* | *** | *112,3* | *2,0* | *512* | *24* |  |  |  | *-2,22* | *-19,9* | 90 |
| *(GW QPA)* | *4* | *28/07/2012* | *12,379* | *17,060* | *906* | *36,81* | *0,50* | *** | *209,0* | *3,4* | *1000* | *48* |  |  |  | *-3,20* | *-32,2* | 37 |
| GW QPA | 1 | 05/08/2012 | 13,477 | 14,707 | 743 | 1,32 | 0,07 | * | <D.L. | 0,7 | 1478 | 132 |  |  |  | 2,91 | 9,3 | 18 |
| GW QPA | 3 | 01/12/2012 | 13,442 | 16,043 | 1085 | 7,46 | 0,48 | * | 0,6 | 6,0 | 1597 | 59 | -9,9 | 60,8 | 0,2 | -4,39 | -30,9 | 46 |
| GW QPA | 2 | 08/12/2011 | 12,670 | 14,790 | 611 | 4,40 | 0,33 | * | <D.L. | NA | 273 | 5 |  |  |  | -2,15 | -16,4 |  |
| GW QPA | 3 | 09/12/2011 | 12,796 | 14,676 | 3490 | 105,90 | 5,96 | * | <D.L. | -0,6 | 217 | 1 |  |  |  | -3,51 | -27,2 | 35 |
| GW QPA | 3 | 09/12/2011 | 12,799 | 14,673 | 1945 | 90,00 | 0,50 | * | <D.L. | 2,6 | 104 | 1 |  |  |  | -5,86 | -46,8 | 36 |
| GW QPA | 3 | 22/07/2012 | 12,800 | 14,679 | 5290 | 228,92 | 0,50 | * | 3,1 | -3,2 | 197 | 10 |  |  |  | -2,93 | -29,1 | 43 |
| GW QPA | 3 | 26/07/2012 | 12,812 | 16,353 | 4480 | 165,74 | 0,50 | * | <D.L. | -4,2 | 196 | 9 |  |  |  | -2,57 | -27,5 | 45 |
| GW QPA | 1 | 13/08/2012 | 10,664 | 15,544 | 404 | 0,27 | 0,01 | * | <D.L. | NA | 2169 | 194 |  |  |  | -2,15 | -21,6 | 22 |
| GW QPA | 1 | 18/11/2011 | 11,647 | 15,271 | 334 | 0,19 | 0,01 | * | 0,1 | 0,6 | 1004 | 44 |  |  |  | -2,68 | -16,6 | 24 |
| GW QPA | 1 | 09/12/2011 | 12,829 | 14,746 | 827 | 0,66 | 0,04 | * | 1,4 | 3,2 | 3905 | 349 |  |  |  | 3,38 | 11,7 | 36 |
| GW QPA | 2 | 08/12/2011 | 12,650 | 14,823 | 227 | 0,34 | 0,02 | * | <D.L. | 4,6 | 2424 | 221 |  |  |  | -2,13 | -14,0 | 40 |
| *(GW QPA)* | *1* | *29/07/2012* | *11,805* | *15,862* | *674* | *40,29* | *0,50* | *** | *22,6* | *2,4* | *469* | *22* |  |  |  | *-4,22* | *-25,4* | 71 |
| GW QPA | 3 | 02/12/2012 | 14,379 | 16,868 | 1615 | 24,56 | 1,39 | * | 5,9 | 4,1 | 320 | 32 | -6,2 | 91,9 | 0,3 | 4,87 | 21,4 | 42 |
| GW QPA | 3 | 02/12/2012 | 13,994 | 16,472 | 1503 | 6,55 | 0,38 | * | <D.L. | 3,6 | 246 | 24 | -5,7 | 76,0 | 0,3 | 5,00 | 23,7 | 53 |
| GW QPA | 2 | 28/07/2012 | 12,533 | 16,747 | 654 | 9,56 | 0,50 | * | 17,3 | 2,7 | 501 | 24 |  |  |  | -2,56 | -23,0 | 69 |
| GW QPA | 2 | 02/12/2012 | 14,077 | 16,539 | 4230 | 122,66 | 7,54 | * | 8,7 | 3,7 | 314 | 16 | -20 | 74,6 | 0,2 | 2,99 | 10,2 | 55 |
| GW QPA | 3 | 09/12/2011 | 12,822 | 14,722 | 1774 | 26,94 | 0,50 | * | 4,8 | 4,3 | 362 | 6 |  |  |  | 1,73 | 0,3 | 37 |
| GW QPA | 3 | 08/12/2011 | 12,894 | 14,629 | 2150 | 50,44 | 0,50 | * | 1,4 | 1,9 | 219 | 6 |  |  |  | 0,28 | -2,8 | 47 |
| GW QPA | 2 | 08/12/2011 | 12,892 | 14,629 | 1167 | 14,90 | 0,50 | * | <D.L. | 3,8 | 264 | 2 |  |  |  | 0,40 | -1,7 |  |
| GW QPA | 3 | 08/12/2011 | 12,890 | 14,634 | 1479 | 22,50 | 0,50 | * | <D.L. | 0,1 | 236 | 6 |  |  |  | 0,18 | -2,1 | 47 |
| GW QPA | 1 | 08/12/2011 | 12,891 | 14,639 | 684 | 0,52 | 0,03 | * | 0,5 | 2,8 | 2076 | 187 |  |  |  | 1,51 | 5,1 |  |
| GW QPA | 1 | 22/07/2012 | 12,891 | 14,639 | 654 | 0,53 | 0,03 | * | 3,4 | -8,0 | 1994 | 185 |  |  |  | 1,56 | 4,2 |  |
| GW QPA | 2 | 08/12/2011 | 12,881 | 14,651 | 1078 | 9,02 | 0,48 | * | <D.L. | 5,6 | 264 | 5 |  |  |  | 1,37 | 3,5 |  |
| GW QPA | 3 | 22/07/2012 | 12,894 | 14,629 | 1763 | 39,33 | 0,50 | * | <D.L. | -4,7 | 192 | 10 |  |  |  | 0,38 | -2,8 | 47 |
| GW QPA | 1 | 08/12/2011 | 12,693 | 14,746 | 184 | 0,39 | 0,02 | * | <D.L. | 1,8 | 1613 | 146 |  |  |  | -1,13 | -9,4 | 25 |
| GW QPA | 2 | 03/12/2012 | 12,980 | 15,524 | 1178 | 20,94 | 1,22 | * | 27,5 | 5,1 | 1830 | 114 | -7 | 90,0 | 0,3 | 6,00 | 26,3 | 20 |
| GW QPA | 3 | 28/07/2012 | 12,501 | 16,667 | 1388 | 62,65 | 0,50 | * | 3,1 | 0,8 | 182 | 9 |  |  |  | -3,00 | -28,6 | 82 |
| GW QPA | 3 | 09/12/2011 | 12,842 | 14,766 | 1639 | 29,66 | 0,50 | * | <D.L. | 4,8 | 212 | 6 |  |  |  | 1,03 | -0,5 | 52 |
| *(GW QPA)* | *3* | *26/07/2012* | *12,549* | *15,899* | *6380* | *390,32* | *0,50* | *** | *598,5* | *0,7* | *220* | *10* |  |  |  | *-2,20* | *-13,6* | 50 |
| GW QPA | 1 | 07/08/2012 | 13,623 | 15,373 | 340 | 3,50 | 0,20 | * | 13,6 | 1,1 | 1128 | 48 |  |  |  | -4,14 | -30,6 | 38 |
| GW QPA | 1 | 03/12/2012 | 13,021 | 15,275 | 1214 | 14,74 | 0,85 | * | 2,3 | 5,9 | 447 | 41 | -4,1 | 87,6 | 0,3 | 4,91 | 19,6 | 16 |
| GW QPA | 3 | 18/11/2011 | 11,962 | 15,155 | 720 | 14,56 | 0,50 | * | <D.L. | 4,5 | 126 | 6 |  |  |  | -3,33 | -24,8 |  |
| GW QPA | 1 | 29/07/2012 | 12,053 | 15,305 | 145 | 4,27 | 0,25 | * | 0,3 | NA | 828 | 60 |  |  |  | -2,89 | -21,2 | 51 |
| GW QPA | 1 | 03/12/2012 | 13,102 | 15,164 | 732 | 0,65 | 0,04 | * | 3,0 | 0,6 | 1472 | 131 | -9,1 | 90,7 | 0,3 | 6,15 | 25,7 | 25 |
| GW QPA | 3 | 08/12/2011 | 12,751 | 14,688 | 2080 | 8,00 | 0,25 | * | <D.L. | 2,1 | 202 | 4 |  |  |  | -2,24 | -19,3 |  |
| GW QPA | 2 | 08/12/2011 | 12,739 | 14,683 | 212 | 0,17 | 0,01 | * | <D.L. | 6,5 | 5088 | 470 |  |  |  | -3,90 | -31,1 |  |
| GW QPA | 2 | 08/12/2011 | 12,735 | 14,680 | 233 | 1,62 | 0,10 | * | <D.L. | 3,2 | 1034 | 98 |  |  |  | -2,16 | -15,1 | 25 |
| GW QPA | 2 | 08/12/2011 | 12,742 | 14,706 | 246 | 0,21 | 0,01 | * | <D.L. | 4,5 | 4348 | 403 |  |  |  | -2,21 | -14,5 |  |
| GW QPA | 2 | 07/08/2012 | 12,974 | 15,716 | 1044 | 10,23 | 0,50 | * | 0,4 | 3,0 | 211 | 10 |  |  |  | 7,79 | 34,3 | 33 |
| *(GW QPA)* | *4* | *01/12/2012* | *13,332* | *15,986* | *588* | *14,60* | *0,86* | *** | *82,3* | *1,6* | *1684* | *47* | *-21* | *79,9* | *0,3* | *-3,65* | *-27,5* | 30 |
| GW QPA | 1 | 18/11/2011 | 10,435 | 15,484 | 262 | 0,47 | 0,03 | * | 0,9 | 5,5 | 1203 | 47 |  |  |  | -2,20 | -20,1 |  |
| GW QPA | 1 | 28/07/2012 | 11,792 | 17,164 | 78 | 0,83 | 0,05 | * | 3,5 | 8,1 | 2734 | 247 |  |  |  | -3,66 | -27,8 |  |
| *(GW QPA)* | *4* | *06/08/2012* | *13,579* | *14,467* | *773* | *38,52* | *0,50* | *** | *210,4* | *-0,8* | *1009* | *49* |  |  |  | *-3,93* | *-29,4* | 37 |
| GW QPA | 3 | 27/07/2012 | 13,157 | 17,422 | 7070 | 301,47 | 0,50 | * | <D.L. | -3,4 | 208 | 10 |  |  |  | -3,12 | -31,7 |  |
| GW QPA | 1 | 29/07/2012 | 11,895 | 15,663 | 428 | 1,57 | 0,09 | * | 35,4 | -5,0 | 2563 | 231 |  |  |  | -6,01 | -40,7 | 47 |
| GW QPA | 1 | 18/11/2011 | 10,664 | 15,544 | 416 | 0,28 | 0,02 | * | <D.L. | 6,4 | 2079 | 99 |  |  |  | -2,22 | -20,3 | 22 |
| GW QPA | 1 | 06/08/2012 | 13,483 | 14,335 | 792 | 29,83 | 0,50 | * | 26,5 | 2,0 | 657 | 31 |  |  |  | 0,33 | -4,2 |  |
| *(GW QPA)* | *3* | *06/08/2012* | *13,543* | *14,895* | *708* | *16,67* | *0,50* | *** | *95,6* | *1,4* | *583* | *27* |  |  |  | *-4,05* | *-31,6* | 34 |
| GW QPA | 3 | 02/12/2012 | 13,748 | 16,497 | 3940 | 207,78 | 12,11 | * | 24,7 | 4,4 | 245 | 25 |  |  |  | -0,28 | -12,2 | 21 |
| GW QPA | 2 | 02/12/2012 | 14,141 | 16,579 | 1405 | 7,80 | 0,45 | * | <D.L. | 5,1 | 265 | 26 | -7,3 | 74,2 | 0,3 | 5,57 | 25,4 | 48 |
| GW QPA | 3 | 03/12/2012 | 12,869 | 15,285 | 2230 | 84,93 | 4,91 | * | <D.L. | 4,4 | 300 | 29 | -6,7 | 54,2 | 0,2 | 1,75 | -0,6 | 52 |
| *(GW QPA)* | *4* | *07/08/2012* | *13,557* | *15,158* | *129* | *2,25* | *0,13* | *** | *22,6* | *2,7* | *2125* | *191* |  |  |  | *-4,44* | *-32,6* |  |
| GW QPA | 2 | 19/11/2011 | 10,073 | 15,583 | 190 | 0,37 | 0,02 | * | <D.L. | 0,6 | 2087 | 48 |  |  |  | -3,81 | -24,4 | 34 |
|  |  |  |  |  |  |  |  |  |  |  |  |  |  |  |  |  |  |  |
| GW DA |  | 04/12/2012 | 12,310 | 15,006 | 708 | 26,52 | *0,5* |  | <D.L. | 0,7 | 21 | 2 | -21 | 0,1 | 0 | -6,56 | -48,1 | 325 |
| GW DA |  | 21/11/2009 | 13,071 | 13,804 | 1071 | 67,64 | *0,5* |  | <D.L. | 1,2 | 17 | 1 |  |  |  | -5,97 | -45,8 | 488 |
| GW DA |  | 21/11/2009 | 13,148 | 13,719 | 1642 | 82,02 | *0,5* |  | <D.L. | NA | 22 | 1 |  |  |  | -5,79 | -45,7 |  |
| GW DA |  | 23/11/2009 | 13,095 | 13,814 | 1726 | 98,71 | *0,5* |  | <D.L. | 0,9 | 15 | 1 |  |  |  | -5,72 | -43,3 |  |
| GW DA |  | 04/12/2012 | 12,571 | 14,933 | 790 | 51,70 | *0,5* |  | 2,954 | 5,9 | 19 | 2 | -7,7 | 0,2 | 0 | -6,47 | -49,1 | 335 |
| GW DA |  | 25/11/2009 | 11,757 | 11,959 | 427 | 4,18 | 0,22 | * | <D.L. | NA | 148 | 8 |  |  |  | -6,89 | -49,9 |  |
| GW DA |  | 25/10/2008 | 13,314 | 12,607 | 975 | 102,93 | *0,5* |  | 0,856 | -1,9 | 34 | 3 |  |  |  | -5,72 | -46,0 |  |
| GW DA |  | 25/10/2008 | 13,320 | 12,610 | 989 | 103,62 | *0,5* |  | 0 | -2,5 | 32 | 1 |  |  |  | -5,74 | -44,4 |  |
| GW DA |  | 22/11/2009 | 12,022 | 13,925 | 856 | 102,79 | *0,5* |  | <D.L. | -3,6 | 13 | 0 |  |  |  | -5,81 | -43,9 |  |
| GW DA |  | 05/03/2014 | 17,917 | 19,094 | 125 | 4,87 | 0,46 | * | 0,004 | NA | 114 | 15 |  |  |  |  |  |  |
| GW DA |  | 05/03/2014 | 17,924 | 19,098 | 195 | 5,37 | 0,53 | * | 0,005 | NA | 108 | 15 |  |  |  |  |  |  |
| GW DA |  | 05/03/2014 | 17,900 | 19,076 | 98 | 2,21 | 0,12 | * | 382 | NA | 171 | 19 |  |  |  |  |  |  |
| GW DA |  | 05/03/2014 | 17,904 | 19,072 | 125 | 2,25 | 0,12 | * | <D.L. | NA | 168 | 19 |  |  |  |  |  |  |
| GW DA |  | 05/03/2014 | 17,923 | 19,100 | 171 | 5,36 | 0,34 | * | <D.L. | NA | 98 | 11 |  |  |  |  |  |  |
| GW DA |  | 23/11/2009 | 12,248 | 13,105 | 419 | 38,07 | *0,5* |  | <D.L. | -0,6 | 14 | 1 |  |  |  | -6,32 | -46,1 |  |
| GW DA |  | 24/11/2009 | 12,493 | 12,786 | 298 | 10,97 | *0,5* |  | 0,265 | 3,2 | 89 | 5 |  |  |  | -6,63 | -48,3 |  |
| GW DA |  | 24/10/2008 |  |  | 752 | 59,31 | *0,5* |  | 1,449 | -1,6 | 61 | 5 |  |  |  | -5,85 | -47,6 |  |
| GW DA |  | 25/10/2008 | 13,294 | 12,452 | 1356 | 116,95 | *0,5* |  | 0 | -2,1 | 25 | 1 |  |  |  | -5,55 | -44,9 |  |
| GW DA |  | 04/12/2012 | 12,420 | 15,028 | 734 | 32,20 | *0,5* |  | <D.L. | 3,5 | 21 | 2 | -20 | 0,0 | 0 | -6,20 | -47,3 | 328 |
| GW DA |  | 25/11/2009 | 11,745 | 12,187 | 395 | 1,67 | 0,09 | * | <D.L. | -4,3 | 142 | 11 |  |  |  | -6,05 | -41,5 |  |
| GW DA |  | 23/11/2009 | 12,688 | 13,511 | 438 | 18,81 | *0,5* |  | <D.L. | -5,8 | 127 | 6 |  |  |  | -6,66 | -47,7 |  |
| GW DA |  | 22/11/2009 | 12,202 | 14,018 | 885 | 51,62 | *0,5* |  | <D.L. | -2,6 | 18 | 1 |  |  |  | -5,95 | -43,9 |  |
| GW DA |  | 22/11/2009 | 11,919 | 13,602 | 589 | 65,00 | *0,5* |  | <D.L. | -0,3 | 13 | 1 |  |  |  | -5,01 | -37,6 |  |
| GW DA |  | 24/11/2009 | 12,115 | 12,831 | 466 | 27,82 | *0,5* |  | <D.L. | NA | 58 | 1 |  |  |  | -6,36 | -46,8 |  |
| GW DA |  | 23/11/2009 | 12,687 | 13,620 | 462 | 18,01 | *0,5* |  | 2,13 | -11,0 | 97 | 5 |  |  |  | -6,56 | -49,1 |  |
| GW DA |  | 24/10/2008 | 14,505 | 13,208 | 917 | 33,40 | *0,5* |  | 0 | -1,3 | 49 | 0 |  |  |  | -6,52 | -46,2 |  |
| GW DA |  | 12/10/2008 | 13,667 | 13,188 | 835 | 57,45 | *0,5* |  | 1,124 | -0,5 | 34 | 3 |  |  |  | -5,92 | -45,1 |  |
| GW DA |  | 12/10/2008 | 13,564 | 12,902 | 742 | 59,14 | *0,5* |  | 1,008 | -2,8 | 39 | 2 |  |  |  | -5,78 | -51,0 |  |
| GW DA |  | 24/10/2008 | 14,258 | 13,107 | 539 | 62,33 | *0,5* |  | 1,287 | -4,1 | 79 | 3 |  |  |  | -5,53 | -47,1 |  |
| GW DA |  | 01/12/2012 | 12,469 | 15,258 | 850 | 57,70 | *0,5* |  | <D.L. | 0,9 | 15 | 2 | -21 | 0,0 |  | -6,41 | -48,3 | 427 |
| GW DA |  | 22/11/2009 | 12,347 | 14,191 | 722 | 21,10 | *0,5* |  | 3,533 | NA | 14 | 1 |  |  |  | -6,91 | -52,7 |  |
| GW DA |  | 22/11/2009 | 12,347 | 14,179 | 697 | 17,96 | *0,5* |  | <D.L. | 2,2 | 11 | 1 |  |  |  | -7,19 | -56,6 |  |
| GW DA |  | 25/10/2008 | 13,763 | 12,606 | 484 | 55,49 | *0,5* |  | 0,372 | -3,2 | 69 | 2 |  |  |  | -5,74 | -47,7 |  |
|  |  |  |  |  |  |  |  |  |  |  |  |  |  |  |  |  |  |  |
| SW - (LB) Chari | | 18/11/2011 | 12,082 | 15,104 | 48 | 0,16 | 0,009 | * | 3,64 | -2,30 | 1353 | 142 |  |  |  | -2,18 |  |  |
| SW - (LB) Chari | | 21/06/2013 | 12,086 | 15,109 | 57 | 0,71 | 0,05 | * | 0,79 | 0,10 | 1271 | 128 |  |  |  |  |  |  |
| SW - (LB) Logone | | 05/12/2012 | 12,070 | 15,054 | 78 | 0,16 | 0,01 | * | 0,40 | 5,30 | 1488 | 152 |  |  |  |  |  |  |
| SW - (LB) Logone | | 21/06/2013 | 12,069 | 15,054 | 55 | 0,70 | 0,04 | * | <D.L. | 0,10 | 830 | 76 |  |  |  |  |  |  |
| SW - (LB) Chari-Logone | | 30/11/2011 | 12,640 | 14,828 | 61 | 0,12 | 0,006 | * | <D.L. | 0,50 | 1567 | 168 |  |  |  | -1,61 |  |  |
| SW - (LB) Chari-Logone | | 04/12/2012 | 12,640 | 14,828 | 65 | 0,15 | 0,01 | * | 0,69 | 6,90 | 1396 | 173 |  |  |  |  |  |  |
| SW - (UB) Logone-Bongor | | 18/11/2011 | 10,275 | 15,366 | 55 | 0,17 | 0,01 | * | 2,21 | 0,80 | 1378 | 139 |  |  |  | -2,58 |  |  |
| SW - (UB) Logone-Moundou | | 19/11/2011 | 8,556 | 16,082 | 54 | 0,16 | 0,009 | * | <D.L. | 4,40 | 1401 | 165 |  |  |  |  |  |  |
| SW - (UB) Taudjile River | | 19/11/2011 | 9,104 | 15,796 | 32 | 0,43 | 0,023 | * | 0,08 | 4,50 | 1276 | 130 |  |  |  | -3,26 |  |  |
| SW - (UB) Nyan River | | 20/11/2011 | 8,314 | 15,796 | 70 | 0,45 | 0,024 | * | 0,42 | 2,20 | 1189 | 119 |  |  |  | -2,62 |  |  |
| SW - (UB) Iro Lake | | 11/04/2015 | 10,135 | 19,411 | 172 | 0,82 | 0,044 |  |  |  | 1147 | 117 |  |  |  |  |  |  |
| SW- (UB) Bahr-Salamat | | 12/04/2015 | 9,976 | 19,379 | 200 | 1,33 | 0,073 |  |  |  | 1172 | 112 |  |  |  |  |  |  |
| SW - (UB) Chari-Sahr | | 12/04/2015 | 9,145 | 18,403 | 94 | 0,92 | 0,05 |  |  |  | 1098 | 106 |  |  |  |  |  |  |
| SW - Lake Chad SP | | 21/07/2012 | 12,961 | 14,599 | 48 | 0,60 | 0,032 | * | 0,20 | 5,40 | 1033 | 96 |  |  |  |  |  |  |
| SW - Lake Chad SP | | 21/07/2012 | 13,057 | 14,642 | 95 | 0,65 | 0,054 | * | 2,10 | 5,40 | 1047 | 35 |  |  |  |  |  |  |
| SW - Lake Chad SP | | 08/12/2012 | 13,006 | 14,564 | 66 | 0,14 | 0,01 | * | 0,39 | 5,90 | 1582 | 198 |  |  |  |  |  |  |
| SW - Lake Chad SP | | 03/03/2014 | 12,958 | 14,595 | 81 | 0,59 | 0,03 | * | <D.L. | 0,10 | 1229 | 106 |  |  |  |  |  |  |
| SW - Lake Chad A | | 05/08/2012 | 13,565 | 14,242 | 364 | 0,90 | 0,048 | * | 0,20 | 0,90 | 763 | 81 |  |  |  |  |  |  |
| SW - Lake Chad A | | 17/06/2013 | 13,466 | 14,710 | 274 | 0,35 | 0,02 | * | <D.L. | 0,10 | 612 | 71 |  |  |  |  |  |  |
| SW - Lake Chad A | | 06/10/2013 | 13,466 | 14,710 | 201 | 0,44 | 0,02 | * | <D.L. | 0,10 | 658 | 77 |  |  |  |  |  |  |
| SW - Lake Chad A | | 17/06/2013 | 13,534 | 14,311 | 345 | 1,62 | 0,10 | * | <D.L. | 0,10 | 189 | 19 |  |  |  |  |  |  |
| SW - Lake Chad A | | 07/10/2013 | 13,534 | 14,311 | 295 | 0,17 | 0,01 | * | <D.L. | 0,10 | 1025 | 146 |  |  |  |  |  |  |
| SW - Lake Chad A | | 17/02/2014 | 13,534 | 14,311 | 277 | 1,69 | 0,11 | * | 0,02 | 0,1 | 69 | 8 |  |  |  |  |  |  |
| SW - KY River | | 12/10/2008 | 13,697 | 13,310 | 81 | 0,61 | 0,033 | * | <D.L. | -4,50 | 692 | 70 |  |  |  | -3,44 | -3,4 |  |
|  |  |  |  |  |  |  |  |  |  |  |  |  |  |  |  |  |  |  |
| rainfall | N'Djamena |  |  |  |  | 0,10 | 0,01 | * |  |  | 374 | 60 |  |  |  |  |  |  |
| rainfall | N'Djamena |  |  |  |  | 0,08 | 0,00 | * |  |  | 374 | 69 |  |  |  |  |  |  |
| rainfall | Cameroon |  |  |  |  | 0,07 | 0,00 | * |  |  | 78 | 52 |  |  |  |  |  |  |
| rainfall | Cameroon |  |  |  |  | 0,06 | 0,00 | * |  |  | 148 | 59 |  |  |  |  |  |  |
| rainfall | Cameroon |  |  |  |  | 0,16 | 0,01 | * |  |  | 136 | 35 |  |  |  |  |  |  |
|  |  |  |  |  |  |  |  |  |  |  |  |  |  |  |  |  |  |  |
|  |  |  |  |  |  |  |  |  |  |  |  |  |  |  |  |  |  |  |
|  | | | |  |  |  |  |  |  |  |  |  |  |  |  |  |  |  |

Table 2. Electrical conductivity, major ions, ^36^Cl/Cl ratios and δ^18^O compositions averages of surface water samples, rainfall samples, Phreatic Quaternary Aquifer Groundwaters divided into four groups and Deep Aquifer Groundwaters. Uncertainties are one standard deviation.

|  | Surface waters | Rainfall | Group 1 | Group 2 | Group 3 | Group 4 | DA |
| --- | --- | --- | --- | --- | --- | --- | --- |
| Number of samples | 59 | 5 | 23 | 17 | 26 | 4 | 33 |
| EC (μS cm^-1^) | 130 +/- 170 | / | 450 +/- 300 | 900 +/- 1000 | 2500 +/- 1800 | 600 +/- 300 | 700 +/- 300 |
| Cl^-^ (mg L^-1^) | 0.6 +/- 1.7 | 0.1 +/- 0.03 | 5 +/- 10 | 15 +/- 30 | **85 +/- 95** | 25 +/- 15 | 45 +/- 35 |
| HCO_3_^-^ (mg L^-1^) | **65 +/- 55** | **/** | **240 +/- 160** | **500 +/- 490** | 520 +/- 230 | 120 +/- 85 | **220 +/- 75** |
| SO_4_^2-^ (mg L^-1^) | 0.3 +/- 8 | 0.1+/- 0.08 | 20 +/- 40 | 85 +/- 170 | **900 +/- 900** | 50 +/- 30 | 10 +/- 150 |
| NO_3_^-^ (mg L^-1^) | 2 +/- 0.8 | 0.2 +/- 0.2 | 6 +/- 10 | 3 +/- 10 | 35 +/- 120 | **130 +/- 95** | **120 +/- 65** |
| Ca^2+^ (mg L^-1^) | **8 +/- 6** | 0.4 +/- 0.3 | **45 +/- 30** | 25 +/- 20 | 110 +/- 100 | 45 +/- 30 | 20 +/- 20 |
| Mg^2+^ (mg L^-1^) | 3 +/- 3 | 0.1 +/- 0.05 | 15 +/- 10 | 10 +/- 10 | 60 +/- 60 | 15 +/- 10 | 10 +/- 7 |
| Na^+^ (mg L^-1^) | 2 +/-0.8 | 0.1+/- 0.01 | 30 +/- 30 | **200 +/- 260** | **430 +/- 340** | 50 +/- 40 | **120+/- 70** |
| K^+^ (mg L^-1^) | 5 +/- 5 | 0.1+/- 0.02 | 10 +/- 10 | 20 +/- 20 | 25 +/- 15 | 20 +/- 20 | 10+/- 5 |
| ^36^Cl/Cl (10^-15^ at/at) | 1100 +/- 350 | 150 +/- 120 | 1500 +/- 900 | 1200 +/- 1500 | 300 +/- 200 | 1400 +/- 500 | 60+/- 50 |
| δ 18O (‰) | -2 +/- 0.7 | / | -1 +/- 4 | 1 +/- 4 | -1 +/- 3 | -4 +/- 0.5 | -6 +/- 0.5 |

Table 3. Average ^36^Cl/Cl ratios and Cl^-^ concentrations in the Chari-Logone River hydrosystem (upstream and downstream), as a function of sampling time. N denotes the number of individual samples used to calculate the average.

| **Date** | **^36^Cl/Cl** | **∆ ^36^Cl/Cl** | **[Cl^-^]** | **∆ [Cl^-^]** | **N** |
| --- | --- | --- | --- | --- | --- |
|  | **(10^-15^at/at)** | **(10^-15^ at/at)** | **(mg/L)** | **(mg/L)** |  |
| **Lower Chari-Logone** |  |  |  |  |  |
| 18-30/11/11 | 1403 | 69 | 0.13 | 0.02 | 2 |
| 7/21/2012 | 1020 | 15 | 0.62 | 0.02 | 2 |
| 04-08/12/12 | 1384 | 52 | 0.152 | 0.006 | 3 |
| 6/21/2013 | 942 | 190 | 0.706 | 0.003 | 2 |
| 3/3/2014 | 1214 | 107 | 0.59 | 0.03 | 1 |
| **Upper Chari-Logone** |  |  |  |  |  |
| 18-20/11/11 | 1266 | 54 | 0.20 | 0.05 | 4 |
| 11-12/04/15 | 1137 | 22 | 0.9 | 0.1 | 3 |
